# A Fab of trastuzumab to treat HER2 overexpressing breast cancer brain metastases

**DOI:** 10.1186/s40164-024-00513-7

**Published:** 2024-04-15

**Authors:** Eurydice Angeli, Justine Paris, Olivier Le Tilly, Céline Desvignes, Guillaume Gapihan, Didier Boquet, Frédéric Pamoukdjian, Diaddin Hamdan, Marthe Rigal, Florence Poirier, Didier Lutomski, Feriel Azibani, Alexandre Mebazaa, Amaury Herbet, Aloïse Mabondzo, Géraldine Falgarone, Anne Janin, Gilles Paintaud, Guilhem Bousquet

**Affiliations:** 1 Université Paris Cité, INSERM, UMR_S942 MASCOT, Paris, F-75006 France; 2 APHP, Hôpital Avicenne, Department of medical oncology, Bobigny, F-93000 France; 3 Université Sorbonne Paris Nord, 99 Avenue Jean Baptiste Clément, Villetaneuse, F-93430 France; 4 Université de Tours, INSERM, U1327 ISCHEMIA EA4245, Tours, France; 5 CHRU de Tours, Centre Pilote de suivi Biologique des traitements par Anticorps (CePiBAc), Tours, France; 6 Pharmacology Department, Tours University Hospital, Tours, France; 7 Université Paris-Saclay, CEA, DMTS, LENIT, Gif-sur-Yvette, SPI F-91191 France; 8 Department of Pharmacy, APHP, Hôpital Avicenne, Bobigny, F-93000 France; 9 Unité de Recherche en Ingénierie Tissulaire-URIT, Sorbonne Paris Nord University, 99 Avenue Jean Baptiste Clément, Villetaneuse, F-93430 France; 10 Department of Anesthesia and Critical Care, APHP, Hôpital Lariboisière, Paris, F-75010 France; 11 APHP, Hôpital Avicenne, Unité de Médecine Ambulatoire, Bobigny, F-93009 France

**Keywords:** HER2 breast cancer, Brain metastases, Fab, Trastuzumab, Pharmacokinetic

## Abstract

Despite major therapeutic advances for two decades, including the most recently approved anti-HER2 drugs, brain metastatic localizations remain the major cause of death for women with metastatic HER2 breast cancer. The main reason is the limited drug passage of the blood-brain barrier after intravenous injection and the significant efflux of drugs, including monoclocal antibodies, after administration into the cerebrospinal fluid. We hypothesized that this efflux was linked to the presence of a FcRn receptor in the blood-brain barrier. To overcome this efflux, we engineered two Fab fragments of trastuzumab, an anti-HER2 monoclonal antibody, and did a thorough preclinical development for therapeutic translational purpose. We demonstrated the safety and equal efficacy of the Fabs with trastuzumab in vitro, and in vivo using a patient-derived xenograft model of HER2 overexpressing breast cancer. For the pharmacokinetic studies of intra-cerebrospinal fluid administration, we implemented original rat models with catheter implanted into the cisterna magna. After intraventricular administration in rats, we demonstrated that the brain-to-blood efflux of Fab was up to 10 times lower than for trastuzumab, associated with a two-fold higher brain penetration compared to trastuzumab. This Fab, capable of significantly reducing brain-to-blood efflux and enhancing brain penetration after intra-cerebrospinal fluid injection, could thus be a new and original effective drug in the treatment of HER2 breast cancer brain metastases, which will be demonstrated by a phase I clinical trial dedicated to women in resort situations.

**Graphical Abstract:**

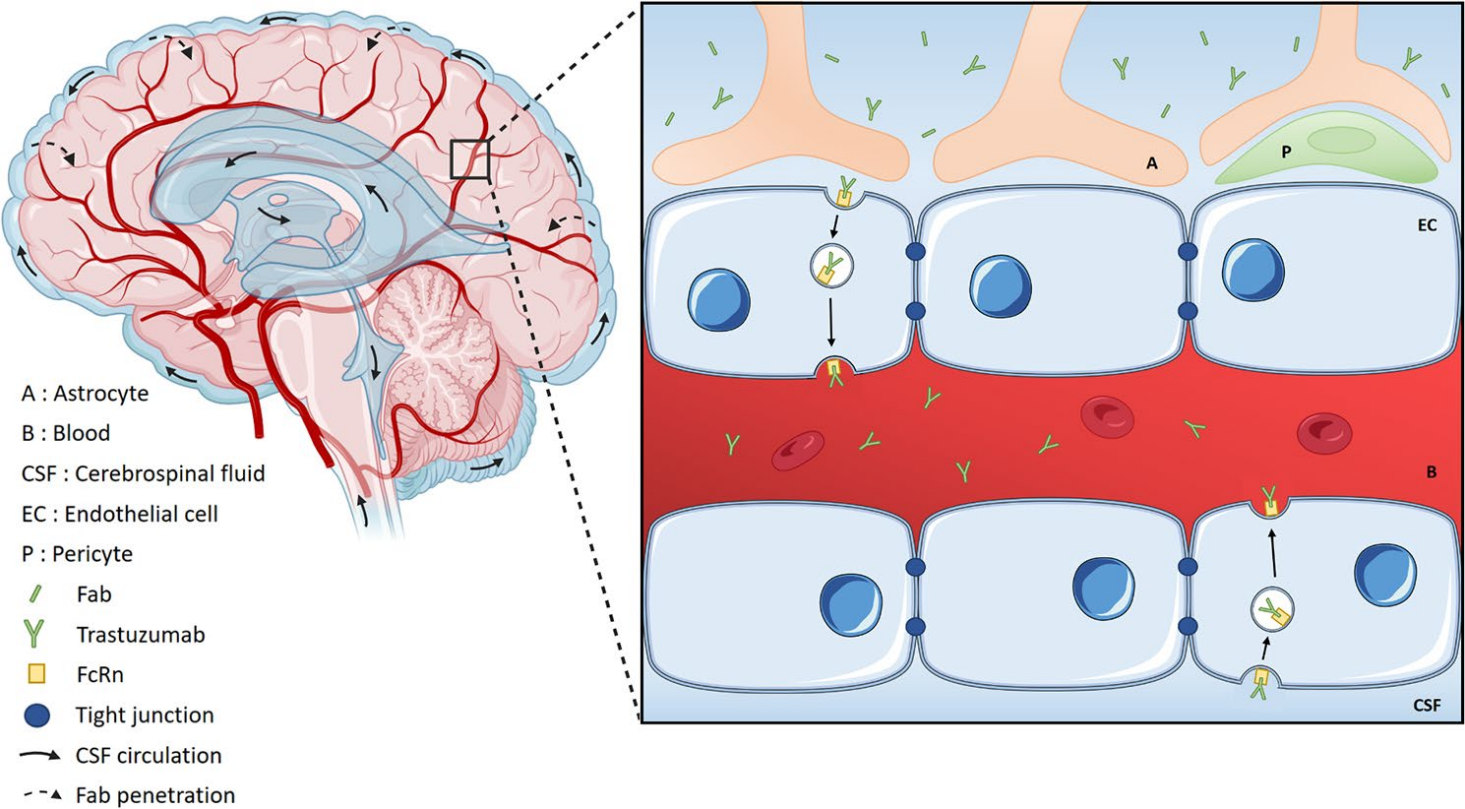

**Supplementary Information:**

The online version contains supplementary material available at 10.1186/s40164-024-00513-7.

## To the Editor

HER2 breast cancer brain metastases are challenging daily practice because of the limited passage of the blood-brain-barrier (BBB) by most treatments [1]. In a pilot pharmacological study, a direct administration of the anti-HER2 antibody trastuzumab into the cerebrospinal fluid (CSF) efficaciously treated HER2-overexpressing breast cancer brain metastases, despite a major brain-to-blood efflux of this therapeutic IgG [2]. We hypothesized that an Fc receptor (FcRn) expressed by endothelial cells of the BBB was responsible for this efflux and that engineering of a Fab antibody would overcome this limitation [3, 4].

Using immunostainings, FcRn receptors were expressed in meningeal and choroid plexus of rats, and mainly at the abluminal side of endothelial cells on human brains (Suppl. Figure 1 A-D). We then did a proof-of-concept study using a commercialized anti-VEGF Fab antibody (ranibizumab) and its corresponding full IgG1 (bevacizumab). We implemented an ultra-sensitive ELISA method to measure ranibizumab concentrations (Suppl. Figure 2 A-B), and an original surgical approach of catheter implantation in the rat cisterna magna to directly inject antibodies into the CSF and perform pharmacokinetic studies (Suppl. Figure 3 A-B). We confirmed the CSF-to-blood rapid clearance of bevacizumab but not of ranibizumab, detected near cerebellar Purkinje cells where VEGF is physiologically expressed [5] (Suppl. Figure 3 C-D).

**Fig. 1:**
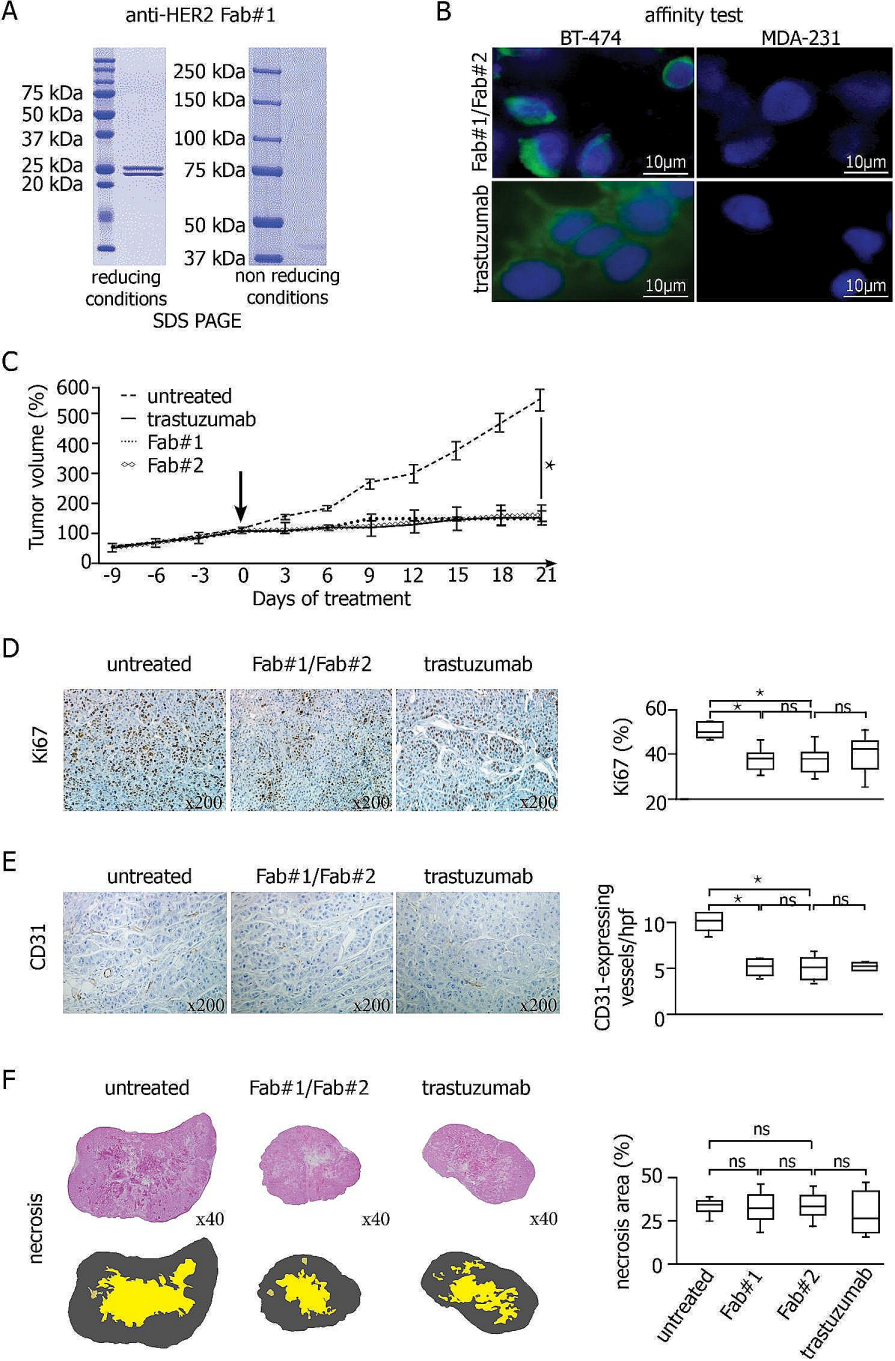
In vitro and *vivo* anti-tumor and toxic effects of anti-HER2 Fab compared to trastuzumab. **(A)** SDS-PAGE with the anti-HER2 Fab#1 under reducing conditions with the light and heavy chains are identified at ~ 25 kDa (left panel), or under non-reducing conditions with the total Fab fragment identified at ~ 40-45 kDa (right panel). **(B)** Affinity test using fluorescence on HER2-overexpressing BT-474 breast cancer cells (left panel) and triple negative MDA-231 breast cancer cells as control (right panel). Cell nuclei are stained in blue (DAPI). Anti-HER2 Fab#2 (top panel) and trastuzumab (bottom panel) are coupled with Alexa Fluor 488 fluorophore (green). **(C) **In vivo antitumor effect of trastuzumab, anti-HER2 Fab#1 and anti-HER2 Fab#2 after intravenous administration (*N* = 20 for each antibody). Tumor growth is expressed in percentage of the tumor volume at day 0 (D0) corresponding to the first day of treatment (black arrow), *P < 0.001. **(D-E-F)** Histological studies of tumors analyzed at Day 21 in untreated mice as control group, mice treated with trastuzumab or with anti-HER2 Fab#2. Proliferation index is assessed with the percentage of Ki67-expressing cancer cells using immunostaining (D), microvessel density with the number of CD31-expressing vessels at high power field (hpf) using immunostaining (E), and necrosis (in yellow) with the percentage of delineated necrotic area on tissue sections (F). ns: not significant, * *P* < 0.001

To target HER2-overexpressing brain metastases, we engineered two Fab of trastuzumab, namely Fab#1 and Fab#2, produced respectively by BIOTEM® and in our research unit (Fig. 1A and Suppl. Figure 4 A). Using fluorescent-labeled antibodies, we confirmed that trastuzumab and the two Fab efficiently bound HER2-overexpressing BT474 cells (Fig. 1B). Flow cytometry showed no significant difference in binding between the three antibodies, and their IC_50_ was identical, of 8 µg/mL. Inhibition proliferation tests at 8 µg/mL showed similar profiles for the three antibodies (Suppl. Figure 4B-D).

In a patient-derived xenograft model of HER2-overexpressing breast cancer (Suppl. Figure 5), intravenous administration of Fab antibodies or trastuzumab at 2 mg/kg/week significantly inhibited tumor growth compared to untreated mice, with no significant difference between the three antibodies (Fig. 1C). Using Ki67 and CD31 immunostainings, treated mice showed a decreased cancer cell proliferation and microvessel density in tumors (Fig. 1D-E), without any increase in necrotic areas (Fig. 1F), in accordance with a well-known neoangiogenesis inhibition of trastuzumab and not a direct cytotoxic effect on tumor endothelial cells [6]. Pharmacological analysis showed higher serum concentrations at 30 min for trastuzumab compared to Fab#1 antibody (Suppl. Figure 6). When we assessed drug toxicity on normal tissues, no histological damage was identified. We assessed biomarkers of cardiac toxicity, the only known toxicity of trastuzumab [7]. *BNP* and *Adrenomedullin* mRNA expressions increased in mice treated with trastuzumab or Fab compared to untreated mice (Suppl. Figure 7 A-B), whereas dipeptidyl-peptidase-3 and cleaved-caspase-3 protein expression was unchanged (Supp. Figure 7 C-D).

**Fig. 2:**
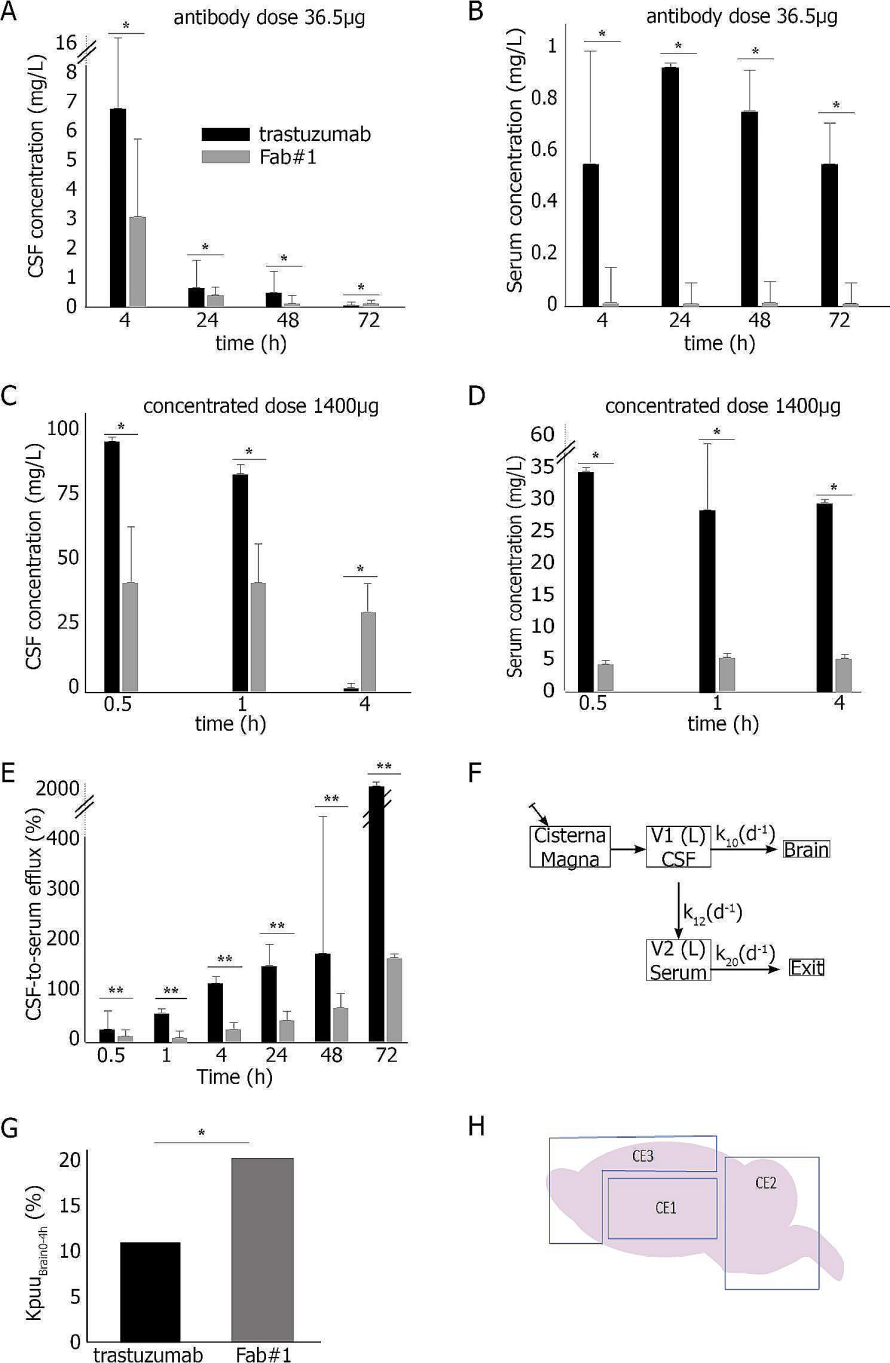
Pharmacokinetic studies after intra-CSF administration of anti-HER2 antibodies in rats. **(A-B)** Pharmacokinetic study with a total dose of 36.5 µg administered for each antibody (*N* = 4 rats for trastuzumab, *N* = 6 rats for Fab#2), in the CSF (A) and in the serum (B). **(C-D)** Pharmacokinetic study with a total dose of 1400 µg administered for each antibody (*N* = 4 rats for trastuzumab, *N* = 3 rats for Fab#2), in the CSF (C) and in the serum (D). **(E)** CSF-to-blood efflux for trastuzumab and anti-HER2 Fab#2 obtained on pooled data (*N* = 8 rats for trastuzumab and 9 rats for Fab#2). **(F)** Schematic of the population pharmacokinetic model used to describe concentrations of each antibody after administration in the cisterna magna. V1: volume of distribution of the CSF compartment, V2: volume of distribution of the serum compartment, k_10_: diffusion constant from CSF to brain; k_12_: diffusion constant from CSF to blood; k_20_: elimination from serum constant. **(G)** Brain concentration assessment of anti-HER2 Fab#2 and trastuzumab from 0 to 4 h after intra-CSF administration. Kp_u, ubrain0−4 h_ = AUC_u_,_brain0−4 h_ /AUC_u_,_csf0−4 h_. **(H)** Schematic dissection of rat brains. CE1 represents deeper brain area, including basal ganglia. CE2 represents posterior area including cerebellum and brainstem. CE3 represents cortical area

After implementing an ultra-sensitive ELISA method to assess low concentrations of the Fab#1 in fluids (Suppl. Figure 8 A-B), two different doses of trastuzumab or Fab#1 were administered in the cisterna magna of rats (36.5 µg first, corresponding to the maximal dose of Fab injected in a volume of 100 µL, and then 1400 µg of a much more concentrated Fab). For the two doses, trastuzumab CSF concentrations rapidly decreased and were almost undetectable at 4 h while it accumulated in the blood. In contrast, and particularly at the higher dose of 1400 µg, CSF concentrations of the Fab#1 remained stable at 4 h with minimal serum detection (Fig. 2A-D). One hour after CSF administration, Fab#1 was almost undetectable in normal lung, liver or kidney (Suppl. Figure 9). When we pooled the 17 animals, the CSF-to-blood ratio steadily increased over time for trastuzumab but remained 10 times lower for the Fab#1 (Fig. 2E). To further investigate the CSF-to-blood efflux, we developed a two-compartment pharmacokinetic model using a population approach (Fig. 5F). We named k_10_ as the diffusion constant from CSF to brain, k_12_ as the diffusion constant from CSF to blood, and k_20_ as the elimination from serum constant. Serum half-life was shorter for Fab#1 compared to trastuzumab (Suppl. Table 1). The k_12_/k_10_ ratio suggested greater brain penetration of Fab#1 compared to trastuzumab. In addition, the calculated partition coefficient Kp_u, ubrain_ [8, 9] from brain samples obtained at euthanasia was 12.3% for trastuzumab and 22.7% for Fab#1 (Fig. 2G). Notably, in deeper brain areas (CE1, Fig. 2H), the Kp_u, ubrain_ for Fab#1 was 2.7 times higher than for trastuzumab, indicating enhanced brain penetration (Suppl. Table 1).

In this preclinical study, we successfully engineered two anti-HER2 Fab antibodies and demonstrated their safety and equal efficacy to trastuzumab for the treatment of HER2 overexpressing breast cancer. Our original pharmacokinetic study with intra-ventricular administration of a Fab antibody demonstrated a limited CSF-to-blood efflux and increased brain penetration. Indeed, our anti-HER2 Fab can penetrate deeper brain parenchyma, which shall be explained by its smaller size and the constant production of CSF by the choroid plexus that creates a pressure directing the fluid flow through the ventricular system to the subarachnoid space [10]. Like for xenobiotics, the Fab probably diffuses *via* the glymphatic system into the cerebral interstitium by convective system, before later elimination into the venous circulation (Graphical abstract) [4]. This innovative approach is promising in view of the development of Fab antibodies for the treatment of brain malignancies but also neurodegenerative diseases [11, 12].

### Graphical abstract

Penetration of Fab in brain parenchyma and efflux of trastuzumab in the blood after intra-ventricular injection. After intra-ventricular injection, the Fab will circulate in the CSF via the glymphatic system into the cerebral interstitium by convective system. The constant production of CSF by the choroid plexus creates a pressure directing the fluid flow through the ventricular system to the subarachnoid space. On the cortical surface of the brain, the cerebral arteries extend into pial arteries running through the subarachnoid and subpial spaces. When they penetrate the brain parenchyma, the pial arteries create a perivascular space filled with CSF known as the Virchow–Robin space, which become continuous with the basal lamina in the deeper brain parenchyma. The Fab will then be capable to fix the HER2 receptor of HER2-overexpressing brain metastatic cell, inhibiting its action. In contrast, when injected, a major part of trastuzumab will fix on FcRn and be eliminated in the venous circulation by transcytosis.

### Electronic supplementary material

Below is the link to the electronic supplementary material.

Supplementary Material 1

Supplementary Material 2

Supplementary Material 3

Supplementary Material 4

Supplementary Material 5

Supplementary Material 6

Supplementary Material 7

Supplementary Material 8

Supplementary Material 9

Supplementary Material 10

Supplementary Material 11

Supplementary Material 12

Supplementary Material 13

## Data Availability

No datasets were generated or analysed during the current study.

## Abbreviations

HER2: Human epidermal receptor 2
Fab: Fragment antigen-binding
BBB: blood-brain barrier
CSF: cerebrospinal fluid
VEGF: Vascular endothelial growth factor
ELISA: Enzyme-linked Immunosorbent Assay
