# Supplementary figures and images for: A Fab of trastuzumab to treat HER2 overexpressing breast cancer brain metastases
Source: Exp Hematol Oncol. 2024 Apr 15;13:41. doi: 10.1186/s40164-024-00513-7 (PMC11017592; doi:10.1186/s40164-024-00513-7)

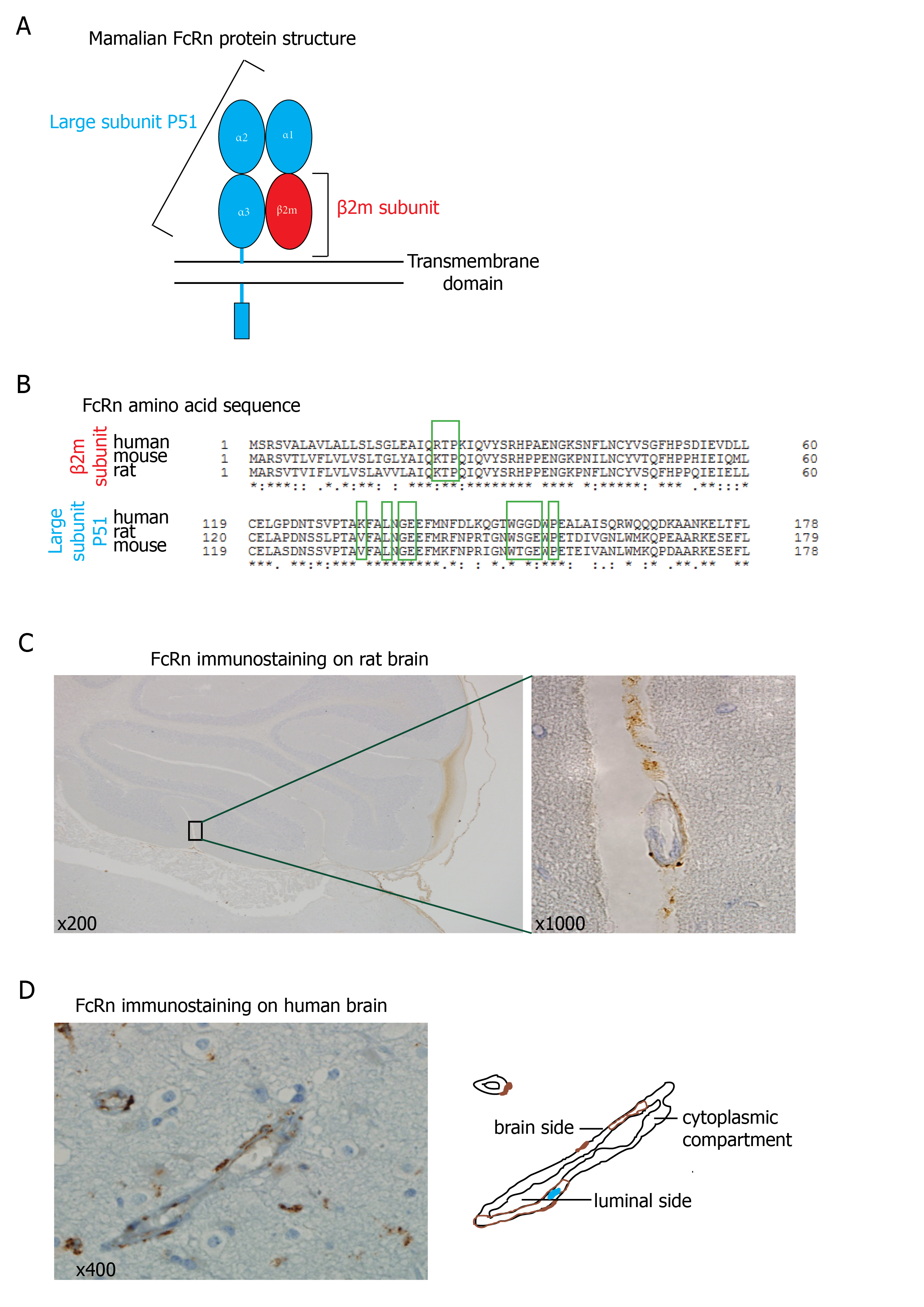

Supplement: Supplementary file 1 — Supplementary Material 1 [file 40164_2024_513_MOESM1_ESM.jpg]

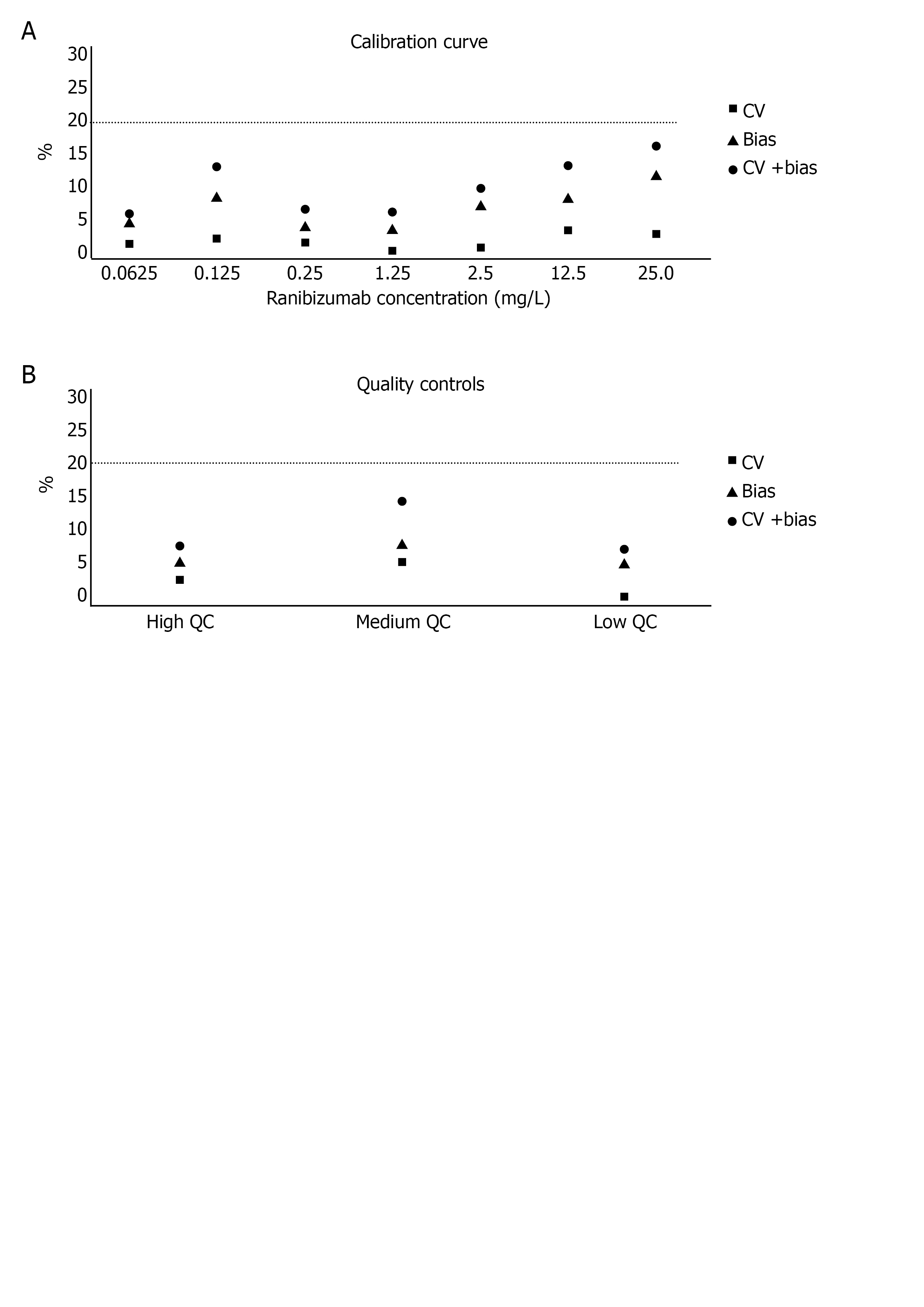

Supplement: Supplementary file 2 — Supplementary Material 2 [file 40164_2024_513_MOESM2_ESM.jpg]

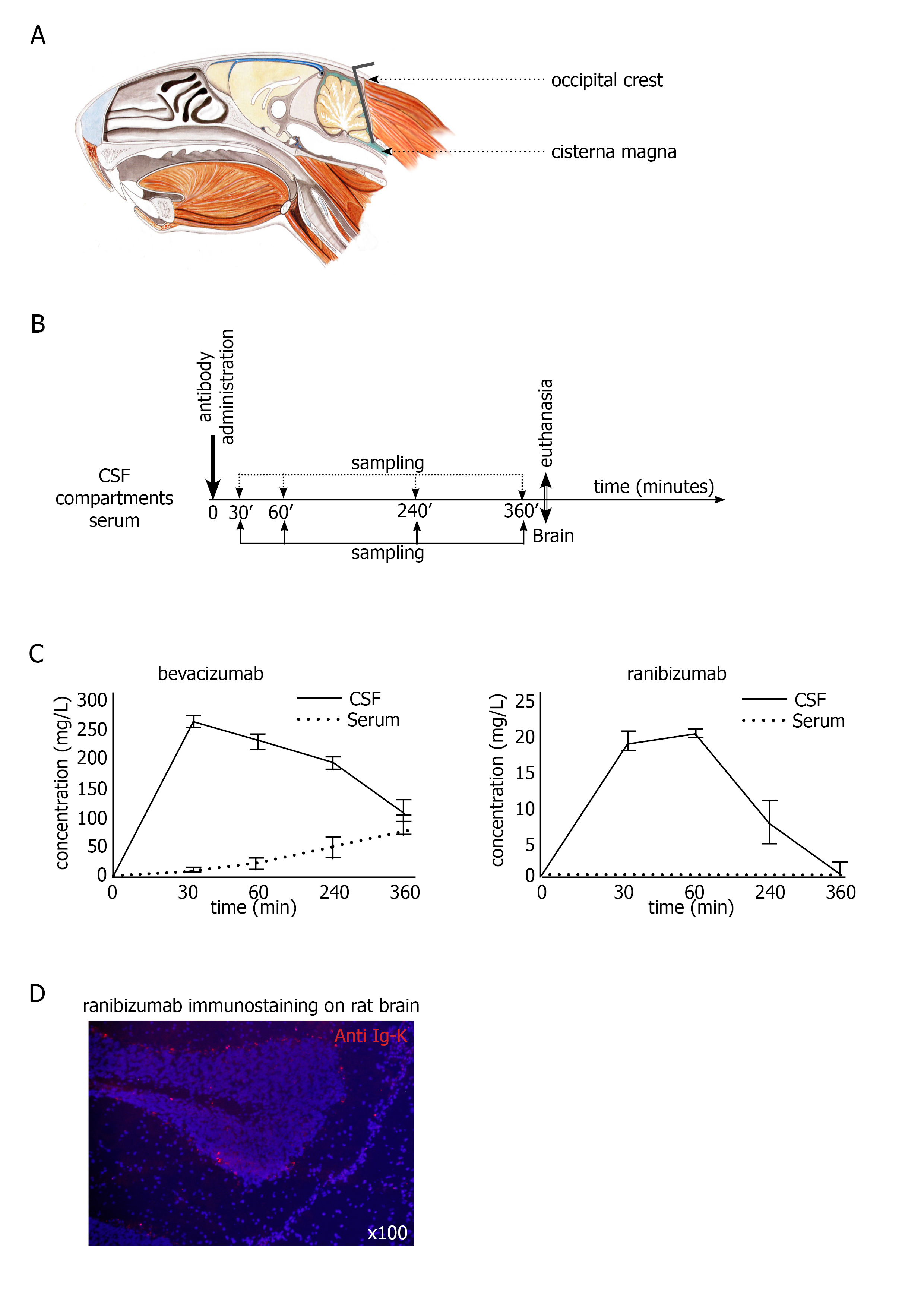

Supplement: Supplementary file 3 — Supplementary Material 3 [file 40164_2024_513_MOESM3_ESM.jpg]

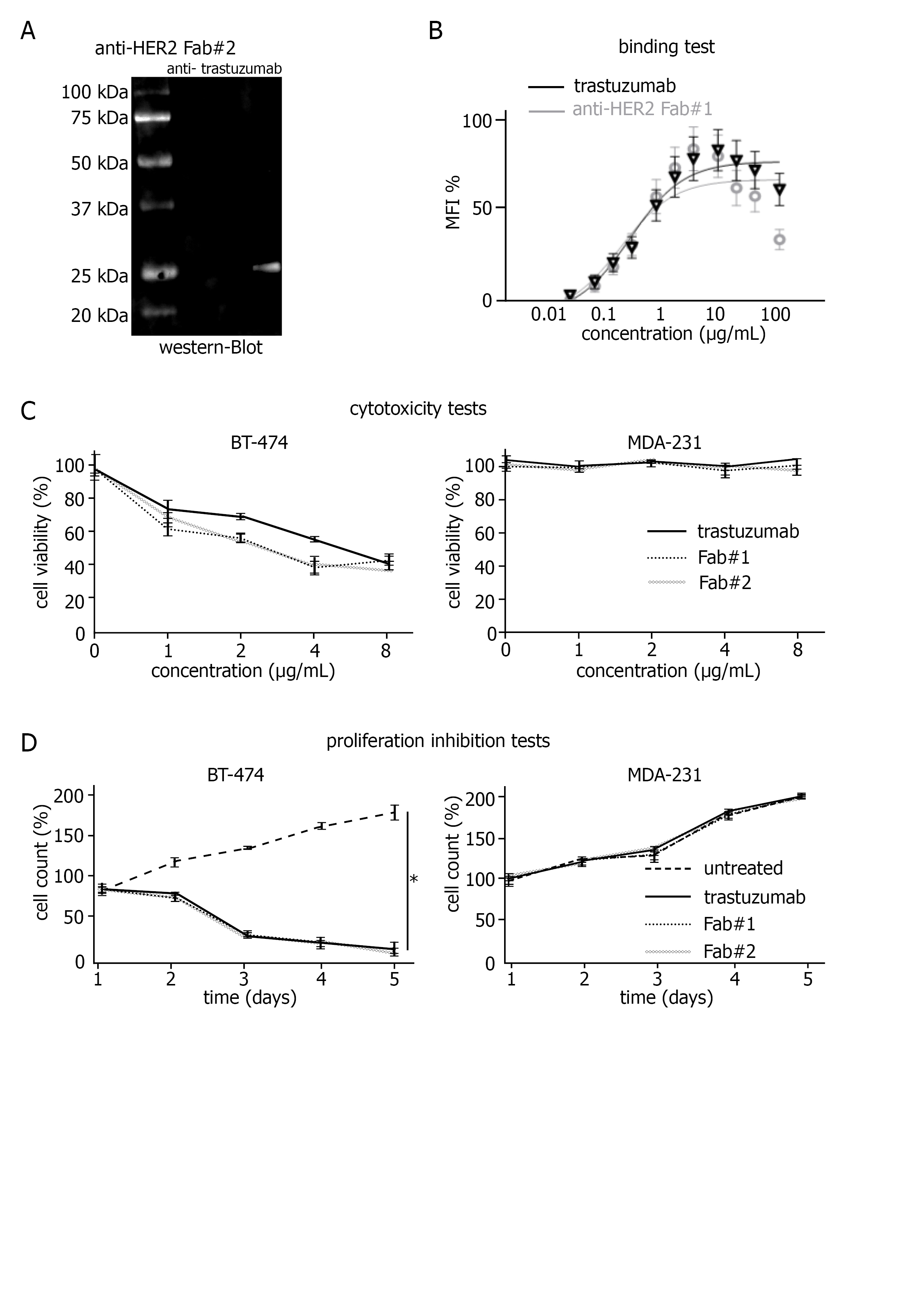

Supplement: Supplementary file 4 — Supplementary Material 4 [file 40164_2024_513_MOESM4_ESM.jpg]

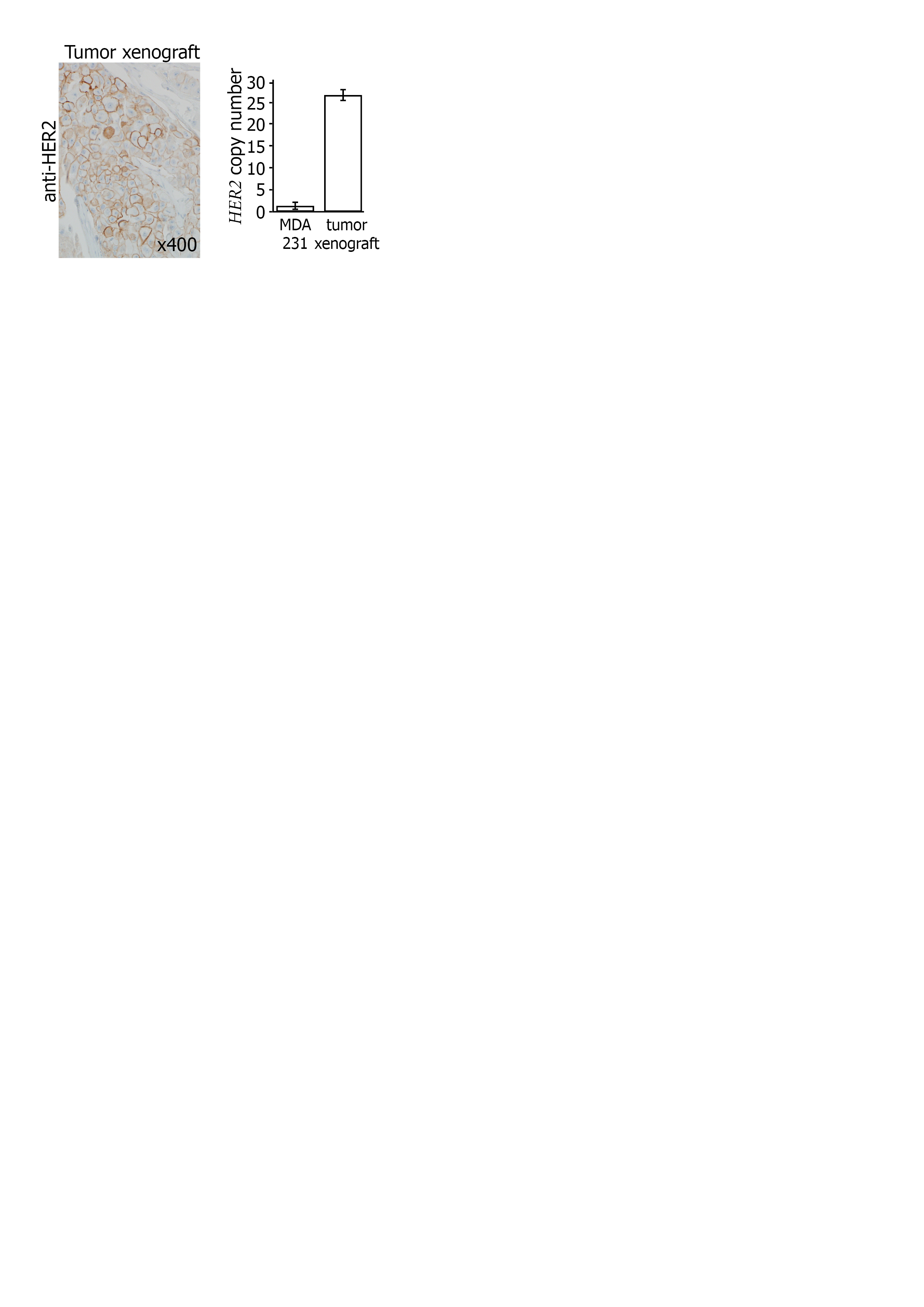

Supplement: Supplementary file 5 — Supplementary Material 5 [file 40164_2024_513_MOESM5_ESM.jpg]

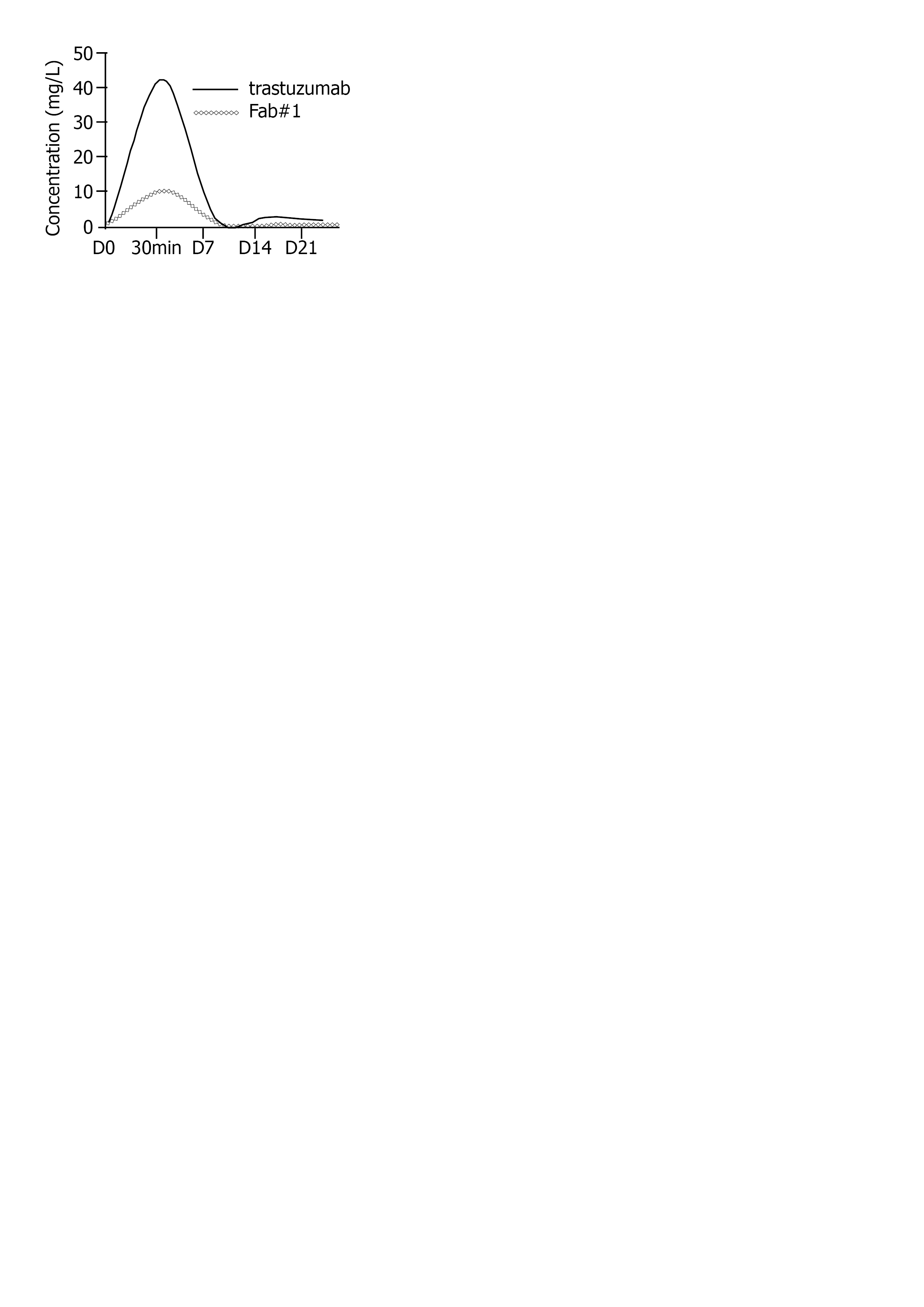

Supplement: Supplementary file 6 — Supplementary Material 6 [file 40164_2024_513_MOESM6_ESM.jpg]

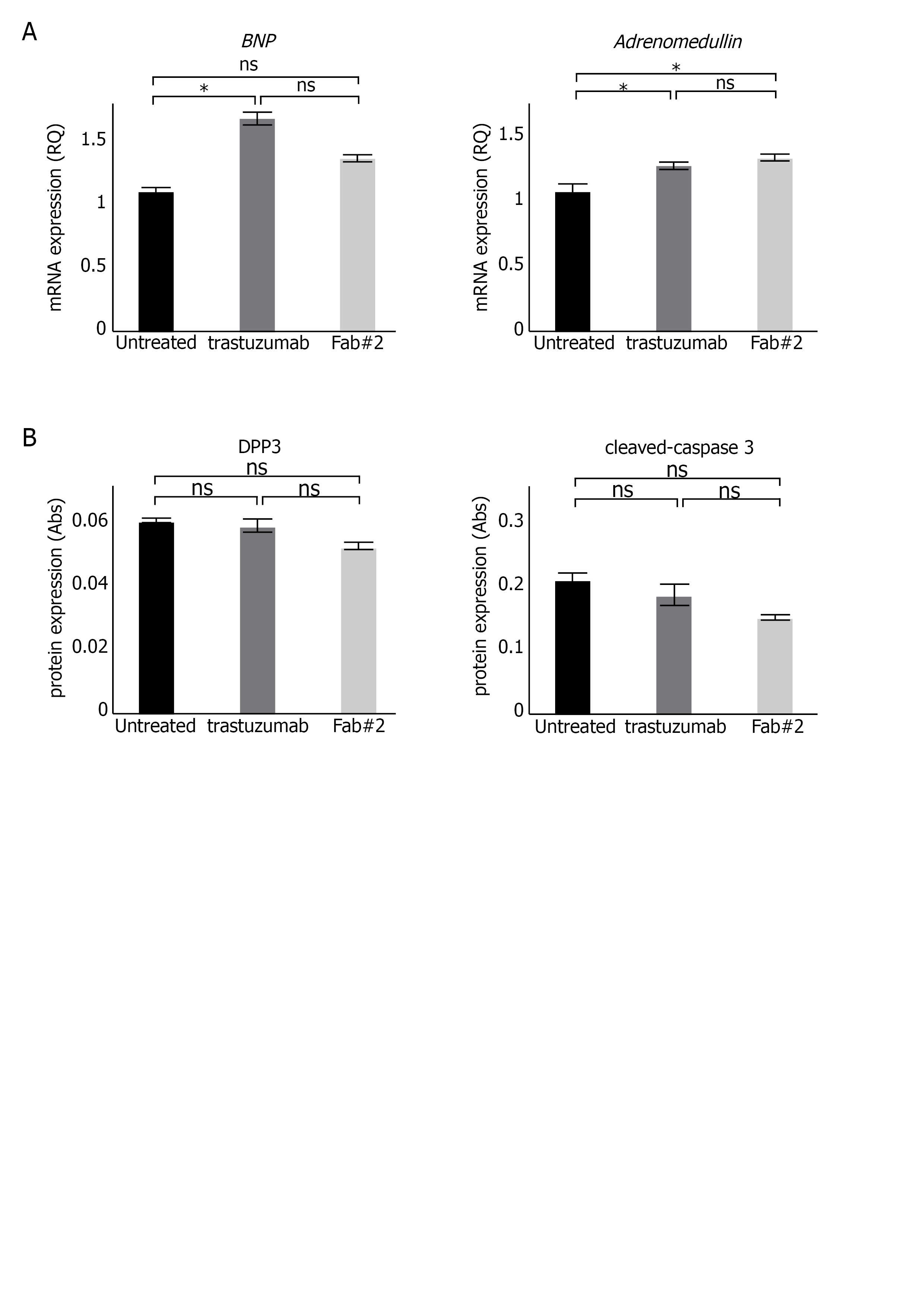

Supplement: Supplementary file 7 — Supplementary Material 7 [file 40164_2024_513_MOESM7_ESM.jpg]

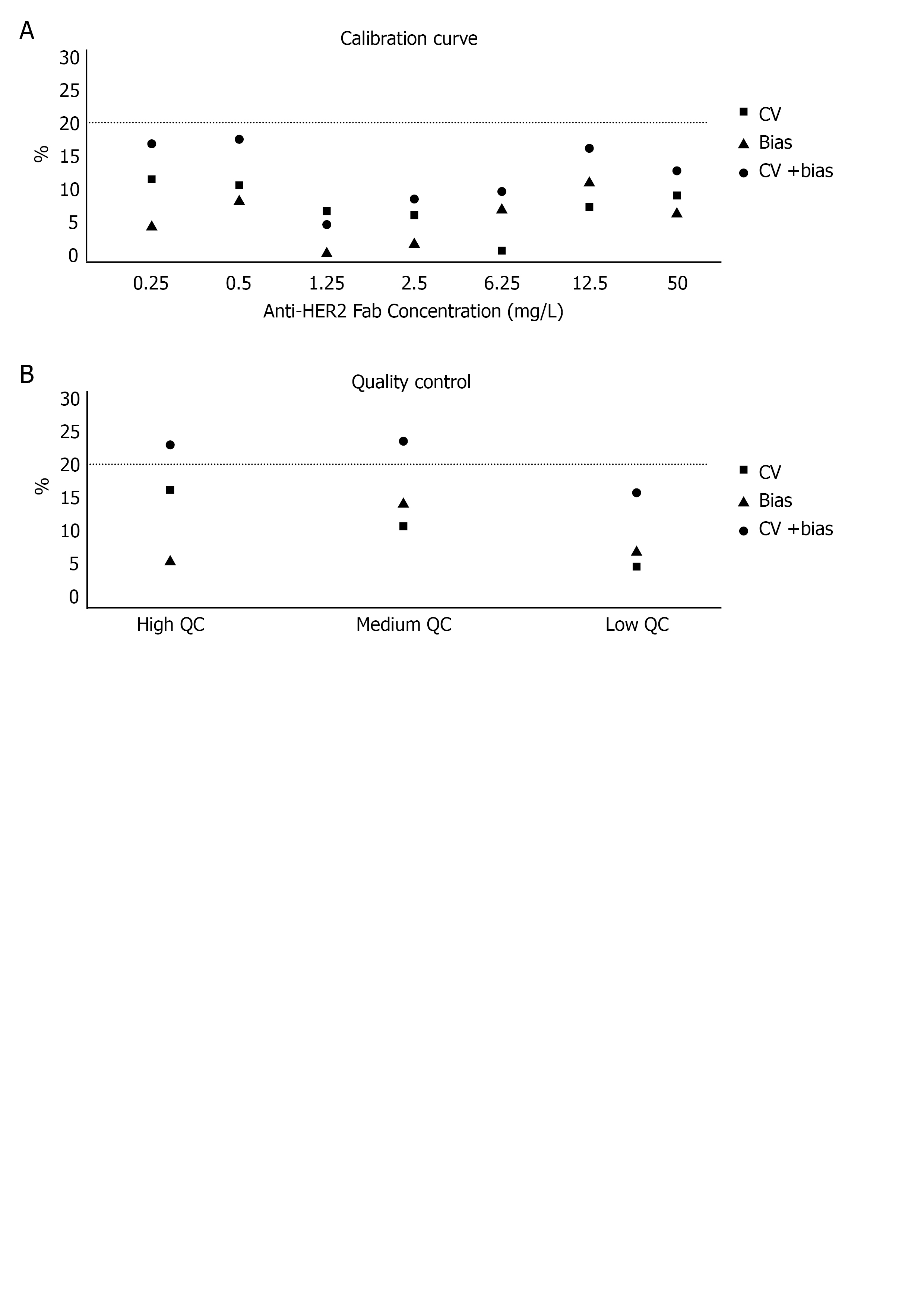

Supplement: Supplementary file 8 — Supplementary Material 8 [file 40164_2024_513_MOESM8_ESM.jpg]

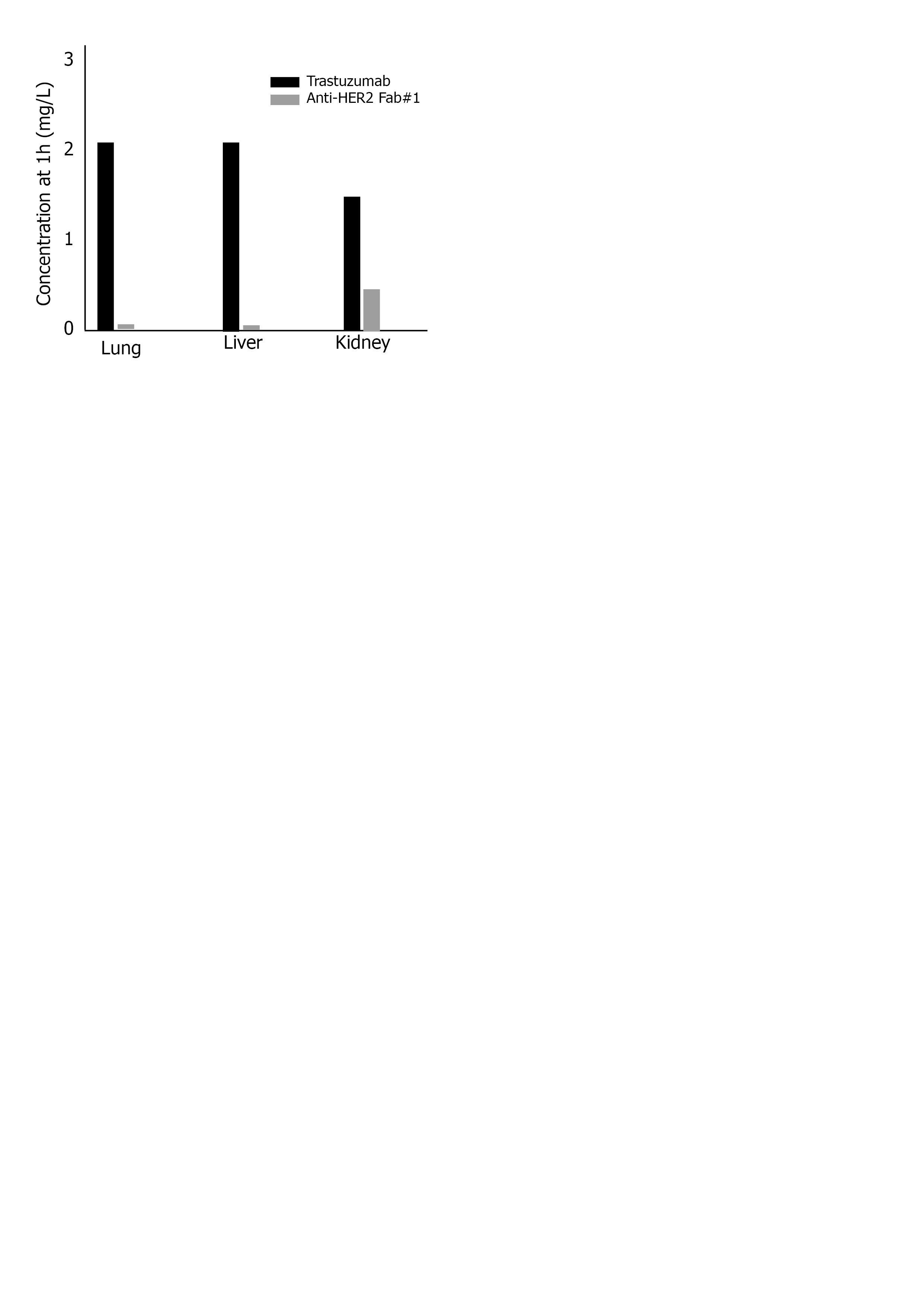

Supplement: Supplementary file 9 — Supplementary Material 9 [file 40164_2024_513_MOESM9_ESM.jpg]
