# Supplementary material for: A Fab of trastuzumab to treat HER2 overexpressing breast cancer brain metastases
Source: Exp Hematol Oncol. 2024 Apr 15;13:41. doi: 10.1186/s40164-024-00513-7 (PMC11017592; doi:10.1186/s40164-024-00513-7)
Supplement: Supplementary file 10 — Supplementary Material 10 [file 40164_2024_513_MOESM10_ESM.docx]

**Supplementary Figure 1**: FcRn structure and expression in human and rat brain.

**(A)** Schematic representation of the mammalian FcRn protein structure. β2m = $\beta2$ microglobulin subunit (in red). **(B)** Alignment of partial amino acid sequences of human, mouse, and rat FcRn using the UniProt Consortium. Residues framed in green are at the binding interface with human Fc fragment of IgG. **(C)** FcRn immunostaining on a rat brain: the left panel at low magnification (X200) shows that FcRn is mainly expressed by arachnoid and pia matter; the right panel at high magnification (X1000) shows a microvessel of the blood-brain barrier and a main FcRn expression on the brain side of the endothelial cell membrane (N = 5 rats analyzed). **(D)** FcRn immunostaining of two microvessels on a human brain at X400 magnification (N = 5 human brains analyzed). The right panel shows the schematic representation of the central venule with the FcRn staining in brown and the endothelial cell nucleus in blue.

**Supplementary Figure 2**: Development of the ELISA method for ranibizumab concentration assessment.

Calibration curve and quality controls. Coefficient of variation (CV), Bias and CV+Bias are expressed in percentages.

**Supplementary Figure 3**: No efflux from brain to blood after intra-CSF injection in rats of the anti-VEGF Fab’2**.**

**(A)** Schematic representation of the surgical procedure to catheterize the cisterna magna. **(B)** Schedule of intra-cerebrospinal injections of bevacizumab or ranibizumab in rats, with CSF, blood and brain sampling. **(C)** CSF and serum pharmacokinetic after intra-CSF administration of bevacizumab (left panel) or ranibizumab (right panel) (N = 5 rats for each antibody). **(D)** Immunofluorescence staining of ranibizumab (red) on a cerebellum circumvolution from a rat brain obtained 360 min after ranibizumab administration. Cell nuclei are stained in blue (DAPI).

**Supplementary Figure 4**: Engineer of anti-HER2 Fab and *in vitro* effects compared to trastuzumab.

**(A)** Western-blot of the anti-HER2 Fab#1 stained with an anti-trastuzumab antibody. The 25 kDa-band corresponds to the fragment of the light chain. **(B)** Binding assessment of anti-HER2 Fab#2 and trastuzumab on HER2-overexpressing BT-474 breast cancer cells. MFI: Mean Fluorescence Intensity expressed in percentage. **(C)** Cytotoxicity test of Fab#1, Fab#2 and trastuzumab on HER2-overexpressing BT-474 breast cancer cells (left panel) and triple negative MDA-231 breast cancer cells (right panel). **(D)** Inhibition proliferation test of Fab#1, Fab#2 and trastuzumab at 8 µg/mL on HER2-overexpressing BT-474 breast cancer cells (left panel) and triple negative MDA-231 breast cancer cells (right panel). * *P* <0.001. All *in vitro* experiments are performed in triplicate.

**Supplementary Figure 5**: Anti-HER2 immunostaining shows HER2 overexpression (3+) of the patient-derived tumor xenograft used for *in vivo* experiments (X400 magnification) (left panel). *HER2* gene copy number using droplet-digital PCR (right panel).

**Supplementary Figure 6**: Pharmacokinetic study from Day 0 (D0) to Day 21 (D21) in serum after intravenous administration of trastuzumab or anti-HER2 Fab#2 (N = 5 for each antibody).

**Supplementary Figure 7**: *In vivo* assessment of cardiac toxicity of anti-HER2 antibodies on the patient-derived xenograft model.

**(A)** *BNP* and *Adrenomedullin* mRNA expression using qRT-PCR and expressed as Relative Quantification (RQ=2^-∆∆Ct^). **(B)** Dipeptidyl-peptidase 3 (DPP3) and cleaved-caspase-3 protein expression using Western-Blot. All experiments are performed in triplicate. * *P* < 0.05.

**Supplementary Figure 8**: Development of the ELISA method for the anti-HER2 Fab concentration assessment.

Calibration curve and quality controls. Coefficient of variation (CV), Bias and CV+Bias are expressed in percentages. QC: Quality Control; High QC 7mg/L; Medium QC 4mg/L; Low QC 0.1mg/L.

**Supplementary Figure 9**: Assessment of tissue concentrations at 1h after intrathecal administration of 1400 µg of trastuzumab or anti-HER2Fab#1 in rats, using ELISA (N = 3 rats for each antibody).
