# Supplementary material for: A Fab of trastuzumab to treat HER2 overexpressing breast cancer brain metastases
Source: Exp Hematol Oncol. 2024 Apr 15;13:41. doi: 10.1186/s40164-024-00513-7 (PMC11017592; doi:10.1186/s40164-024-00513-7)
Supplement: Supplementary file 11 — Supplementary Material 11 [file 40164_2024_513_MOESM11_ESM.docx]

Table 1: Mean population pharmacokinetic parameters after intra-CSF administration of anti-HER2 antibodies in rats and partition coefficients in rat brains.

|  | Trastuzumab | Fab#1 |
| --- | --- | --- |
| V1 (mL) | 0.314 | 0.059 |
| V2 (mL) | 17.8 | 5.83 |
| k_10_ (day^-1^) | 0.261 | 2.34 |
| k_12_ (day^-1^) | 60.6 | 237 |
| k_20_ (day^-1^) | 0.372 | 56 |
| k_12_/k_10_ | 232 | 101 |
| Kpu,u_brain,0-4h_ (%) | 12.33 | 21.51 |
| Kpu,u_CE1,0-4h_ (%) | 8.65 | 23.82 |
| Kpu,u_CE2,0-4h_ (%) | 14.70 | 19.67 |
| Kpu,u_CE3,0-4h_ (%) | 13.64 | 21.04 |

V1: volume of distribution of the CSF compartment, V2: volume of distribution of the serum compartment, k_10_: diffusion constant from CSF to brain; k_12_: diffusion constant from CSF to blood; k_20_: elimination from serum constant. Kpu: partition coefficients from CSF to brain from 0 to 4h after injection in the total brain (Kpu,u_brain,0-4h_), in CE1, deeper area (Kpu,u_CE1,0-4h_), in CE2, posterior area (Kpu,u_CE2,0-4h_ ), and in CE3, superficial area (Kpu,u_CE3,0-4h_).
