# Supplementary material for: A Fab of trastuzumab to treat HER2 overexpressing breast cancer brain metastases
Source: Exp Hematol Oncol. 2024 Apr 15;13:41. doi: 10.1186/s40164-024-00513-7 (PMC11017592; doi:10.1186/s40164-024-00513-7)
Supplement: Supplementary file 12 — Supplementary Material 12 [file 40164_2024_513_MOESM12_ESM.docx]

**Methods**

**FcRn expression in rat brain**

FcRn is a transmembrane protein composed of two subunits: a large transmembrane alpha chain with 3 extracellular domains (α1, α2, α3), also called the subunit P51, and the β2 microglobulin subunit (1) (Suppl.Fig.1A). FcRn is ubiquitously expressed across different cells, tissues and species, including endothelial cells of the mammalian BBB (1). Between human and rat, the homology of amino acid sequence is 67.1% for the entire protein composed of 484 amino acids (Suppl.Fig.1B). It is 64.6% for the P51 subunit and 69.7% for the β2microglobulin subunit. In contrast, the IgG binding site (green box on Suppl.Fig.1B) contains amino-acids that are highly conserved between species (2).

Brains from five untreated *Rattus norvegicus* (Charles River, France) aged of 6 weeks were carefully removed after euthanasia, formalin-fixed, paraffin-embedded and cut into 5 µm-thick sections. FcRn expression was assessed with an indirect immunoperoxydase method, using a goat anti-rat FcRn as the primary antibody (R&D Systems; AF6775), and a biotinylated rabbit anti-goat IgG as the secondary antibody (Vector Laboratories, Inc., Burlingame, CA). Standardized controls were the absence of primary antibody and the use of an irrelevant primary antibody of the same isotype. Tissue sections were analyzed under an Olympus AX 70 microscope with a 0.344-mm^2^ field size at X400 magnification. Analyses were performed by two pathologists (GB, AJ).

**Proof of concept with anti-VEGF antibodies**

A first step of proof of concept was necessary to validate the absence of efflux of Fab fragments, using two commercialized anti-VEGF antibodies, bevacizumab (full-length IgG, 149 kDa) and ranibizumab (Fab, 48 kDa).

***Enzyme-linked Immunosorbent Assay (ELISA) procedures for bevacizumab and ranibizumab concentration assessment in serum, brain and cerebrospinal fluid***

Bevacizumab and ranibizumab were provided by the pharmacy of Avicenne Hospital. For ranibizumab concentration assessment, we developed a protocol based on the procedure implemented by Ternant *et al* to assess bevacizumab serum concentrations in patients (3). Microtiter 96-well plates were prepared by incubating 100 $\mu$L VEGF_165_ at a concentration of 0.25 mg/L in the coating buffer (1 mol/L carbonate–bicarbonate buffer) overnight at 4°C. The plates were washed four times with PBS containing 0.05% Tween 20. The remaining protein-binding sites were blocked by 2 hours incubation at room temperature with 200 $\mu$L blocking buffer (PBS containing 1% BSA). Plates were washed 4 times and 100 $\mu$L of 1:100 diluted standards, quality controls (QCs), and samples were added. After incubating the plates for 2 hours at 37°C and following a new washing round, 100 $\mu$L of anti-human secondary antibodies (anti-human kappa light chain, Sigma 1:3500 diluted) coupled with peroxidase, diluted in 1% PBS-BSA was added to each well. After 1 hour at room temperature followed by washing, 100 $\mu$L OPD (prepared by dissolving tablet sets in 20 mL distilled water) was added and the reaction was allowed to develop at room temperature in the dark. The color reaction was stopped by adding 50 $\mu$L of 2 mol/L sulfuric acid per well. Reading was performed at two wavelengths (490 and 630 nm) using an ELISA plate reader (CLARIOstare, BMG Labtech). The absorbance at 620 nm corresponds to the background signal linked to the plate and was subtracted from the absorbance at 492 nm.

For the accuracy of quality control and standard curve determination, we used values with the better inter-day accuracy, determined as coefficient of variation <20% (reflect of the reproducibility) and bias from expected concentrations <20%. After their determination, each quality control and standard points were tested again 12 times to ensure that coefficient of variation was <20% and bias from expected concentrations was <20% (validation step). The limit of detection was calculated by assaying 12 replicates of blank samples at 3-times of the mean standard deviations. The lower limit of quantification corresponded to the lowest calibration standard. The upper limit of quantification was defined as the highest amount of ranibizumab that could be quantified with standard deviation and relative error *<*20%. To ensure that samples can be diluted in case of peak concentrations outside of the standard curve, we tested the reproducibility of a series of increasing dilutions, corresponding to dilution linearity.

For bevacizumab we used an ELISA procedure already validated for clinical practice (3-6). We used an anti-human IgG Fc specific as secondary antibody, coupled with peroxidase (Sigma).

***Implantation of catheter into the cisterna magna of rats, preparation of samples***

We developed our own method of cisterna magna catheterization across the occipital crest. We used a special surgical setup for minimally invasive repeated sampling and injections that can be used for a prolonged time. Our procedure was developed in *Rattus norvegicus* (Charles River, France) aged of 6 weeks. During the whole experiment, rats were maintained in environment adapted to their housing conditions. The use of rats as well as catheterization of the cisterna magna has been approved by the Ministry of Research (APAFIS#17189-2018101814041264), and by the Ethics Committee on animal experiments.

The rat is first anesthetized with a cocktail of isofluorane 3% and a mixture of buprenorphine at 0.3 mg/mL (0.05 mg/kg), ketamine at 100 mg/mL (90 mg/kg), xylazine at 100 mg/mL (10 mg/kg). It is then placed on a stereotactic frame with the head positioned in order to achieve a flat skull. On sterile conditions, the operating area is shaved and cleaned using dermic betadine. Skin is incised on 2-3 cm length from the dorsal midline of the skull to the occipital crest. Using blunt scissor, skin is disjoined from the skull. The skull is then exposed by gently scraping the periosteum with a scalpel. Using the stereotactic frame, a 0.7 mm hole is drilled into the interparietal bone, 1 millimeter ahead of the occipital crest on the sagittal midline, at an angle of 60-70° from the horizontal plan, in the caudal direction, at 5 mm depth. Catheter (SAI infusion Technologies) is then placed into the hole and fixed at the skull with a drop of tissue glue (histoacryl, Braun). Cutaneous plan is then sutured, and the end of the catheter is placed under the skin.

For this step, the objective is to analyze the intrathecal pharmacokinetics after intrathecal injection of ranibizumab or bevacizumab antibodies. Two antibody solutions were prepared. Fourteen μg of bevacizumab and ranibizumab solutions were diluted in saline (0.9%) to achieve a total volume of 100 µL. Each solution was injected into the cisterna magna using the catheter, in a total of 10 rats (5 rats per group). Then, 50 μL of cerebrospinal fluid and blood were taken at 0 (before injection of the solution), 30 minutes, 60 minutes, 180 minutes and 240 minutes after injection. Once collected, the blood tube was centrifuged at 10,000 RPM at 4°C for 10 minutes to isolate the serum. The cerebrospinal fluid and serum were then analyzed with the developed ELISA technique (described in the result section).

**Engineering of anti-HER2 Fab fragments**

Two anti-HER2 Fab fragments were engineered for this study, using the same methodological approach: one specifically in our research team, and the second in collaboration with BIOTEM®.

For the Fab fragment we have engineered (Fab#1), light chain and heavy chain of the Fab were synthesized separately and then assembled into chinese hamster ovary (CHO) cells. The light chain was synthesized in pcDNA3.2 vector, using the sequence corresponding to the light chain of trastuzumab and the heavy chain was synthesized in pFUSEss-CHIg-hG1 vector (Invitrogen, USA). A stop codon was added at the end of each sequence to avoid adding other elements to the sequence of interest.

Sub-cloning in expression vector was first realized in *E.Coli XL1-Blue bacteria*. *E.Coli* were first transformed with genes or vectors separately by heat shock at 42°C for 30 seconds followed by 2 minutes on ice, to amplify them. Bacteria were then incubated in a rich SOC medium (Invitrogen, USA) for 1 hour at 37°C at 230 RPM and then cultured in a liquid environment containing the appropriate selection antibiotic. The tubes were incubated at 37°C overnight. For each gene or vectors, plasmid DNA was recovered with the Wizard Plus SV Minipreps DNA Purification System Kit (Promega, USA), which allows the production of a clarified lysat and the purification of plasmid DNA. A PCR was performed with the purified plasmids. The products were digested with Dpn1 (New England BioLabs, France) for 2 hours in order to get rid of bacterial DNA. Products were purified again before being controlled on 1% agarose gel (Invitrogen, USA).

Once the size of genes and vectors was controlled by gel migration, vectors and genes were treated 2 hours with T4-DNA polymerase (New England BioLabs, France) to generate blunt ends. *E.Coli* were transformed by heat shock with the gene of the Fab light chain or the heavy chain, their vector and its specific primers. Each gene and its corresponding vectors were assembled with specific primer. A colony-based PCR was performed. Each colony collected was amplified and PCR products were controlled on 2% agarose gel (Invitrogen, USA). For each product the plasmid DNA was recovered, sequenced, and analyzed by alignment with the expected sequence.

Insertion in synthesis vector (CHO) was performed to synthesize the Fab. The CHO cells were seeded 24 hours before transfection with 70 to 90% confluence in 6 wells plates. The two plasmids (the light chain and heavy chain) were placed together in the culture medium, with a transfection agent (Sigma-Aldrich) at a 3:1 ratio. They were incubated for 15 minutes at 20°C before being added to each well containing CHO cells. After 24 hours, the antibiotics were added to achieve 50% mortality of the cells and then gradually increased to 100% mortality of non-transfected cells. After several days, the culture medium was removed and purified by column chromatography or concentrated (Vivaspin Turbo 15R, Sartorius). A SDS-PAGE gel was then performed to ensure the weight of the Fab. A Western Blot was performed with anti-trastuzumab antibody (R&D SYSTEM). To control the amino-acid sequence of the Fab fragments by tandem mass spectrometry (MS/MS), the protein bands corresponding to the to the light and heavy chains of the Fab were excised from the SDS-PAGE under reducing conditions and proteins were in-gel digested as previously described (7). Peptide samples were analyzed with a QTOF mass spectrometer (Impact HD, Bruker) equipped with the CaptiveSpray ion source (Bruker). The QTOF was coupled to a nano liquid chromatography (Ultimate 3000, ThermoFisher Scientific) running with two buffers: 0.1% formic acid in water and 0.1% formic acid in 80% acetonitrile. Chromatographic separation was carried out on a C18 reverse phase column (75 μm, 150 mm, 120 Å Wide Pore, Bruker) with a gradient elution at a flow rate of 300 nL/min during 60 min. The system was operated with automatic switching between MS and MS/MS modes using a data-dependent acquisition (DDA) method for peptide fragmentation. MS/MS spectra were then processed with the Data Analysis software (Bruker). In a first step, peptides have been identified automatically using the MASCOT software (Matrix Science, London, UK) and the SwissProt database (www. expasy.org) with the following parameters: carbamidomethylation for cysteine residues; potential oxidation for methionine residues; tolerance on mass measurements of 20 ppm in MS mode and 0.1 Da in MS/MS mode; enzymatic cleavage by trypsin with one missed cleavages allowed. The species of origin was restricted to the Mammalia. In a second step, peptides not automatically identified, were then manually sequenced on the basis of their MS/MS spectra using the annotate tool in the Data Analysis software (Bruker).

For the Fab fragment synthesized in collaboration with BIOTEM® (anti-HER2Fab#2), the synthetic genes encoding the anti-HER2 Fab light and heavy chains were designed and controlled by sequencing, then subcloned in BIOTEM® proprietary vector and transfected in CHO cells. The supernatant was then purified by affinity chromatography and controlled by size exclusion chromatography.

**Validation of anti-tumoral effect of Fab anti-HER2 fragments compared to trastuzumab**

***Human cancer cell lines***

Two breast cancer cell lines were used, BT474 with HER2 overexpression and MDA-MB-231 which does not overexpress HER2. The cell lines were obtained from ATCC. These cells were cultured at 37 °C under normoxic conditions (20 % of O_2_ and 5 % of CO_2_) in RPMI-1640 medium supplemented with 10 % of fetal calf serum and 1 % antibiotics.

***In vitro affinity and cytotoxicity of anti-HER2 Fab antibodies***

The ability of trastuzumab or Fab anti-HER2 fragments to efficiently bind to HER2 membrane receptors was assessed on the BT-474 and MDA-MB-231 cell lines. The two cell lines were grown separately on culture slides (BD Falcon™). Five micrograms of commercial trastuzumab (Roche) or anti-HER2 Fab fragments coupled with Alexa Fluor 488 fluorophore (using APEX™ Alexa Fluor™ 488 Antibody Labeling Kit, Invitrogen) were diluted in 300 µL of PBS and incubated for 1 hour with each type of human cancer cell line. Then, the PBS was removed and the cells were rinsed to remove unbound antibodies. The cells were fixed in acetone at 4°C, the nuclei were stained with DAPI (Vector Laboratories, Vectashield, H-1200) and fluorescence staining was observed at X400 magnification. The experiment was conducted five times independently, and a minimum of 100 cells were analysed.

For saturation binding experiment, BT474 cells were cultured in RPMI1640 supplemented with 10% fetal calf serum, 2 mM L-glutamine, 100 U/mL penicillin and 100 µg/mL streptomycin. Cells were maintained at 37°C in a humidified atmosphere of 5% CO_2_. All media and cell culture supplements were from Life Technologies. Collected cells were seeded (300,000 cells/tube). Cells were incubated overnight at 4°C with 600 µL of D-phosphate-buffered saline (PBS) supplemented with 0.1% BSA and 5% normal goat serum (NGS, Life Technologies) and containing increasing concentrations of trastuzumab or anti-HER2Fab antibodies. After two washes with 1000 µL of ice-cold D-PBS, and in order to reveal the binding, cells were incubated for 4h at 4°C in the dark with Goat anti-Human Kappa Light Chain Secondary Antibody, FITC (Invitrogen catalog #A18854) in 300 µL of D-PBS supplemented with 0.1% BSA and 5% normal goat serum. After two washes with 1000 µL of ice-cold D-PBS, fluorescence was measured using a FACSCalibur (BD BIOSCIENCES). Then mean fluorescence intensity (MFI) of samples was then determined by cytometer software. The MFI results were analyzed and curves were fitted using GraphPad Prism with the adequate dose-response and IC50±0.1 SD values were determined.

To assess anti-HER2 Fab fragment cytotoxicity, BT474 cells were seeded in 96-well tissue culture plates at a density of 5.10^3^ cells per well. After 24 hours of incubation, the cells were exposed to increasing concentrations of anti-HER2 Fab fragment or trastuzumab (0 to 8 µg/mL) for 72 additional hours. Cell viability was determined by the colorimetric conversion of yellow, water-soluble tetrazolium MTT (3-[4, 5-dimethylthiazol-2-yl]-2,5-diphenyl-tetrazolium-bromide; Sigma), to purple, water-insoluble formazan. After incubation for 4 h at 37 °C with 0.4 mg/mL of MTT, the cells were placed in 0.1 mL of DMSO, and the absorbance was measured at 560 nm using a CLARIOstar® plate reader (BMG LABTECH). Experiments were performed in triplicate, untreated cells being used as positive controls, and drug-containing medium without cells as a negative control. Results were expressed as percent of cell viability compared to untreated cells.

To assess anti-HER2 Fab fragment inhibition proliferation effect, BT474 cells were seeded in 96-well tissue culture plates at a density of 5.10^3^ cells per well. Then, 8 µg/mL of anti-HER2 Fab or trastuzumab were added and cells were counted each day for 5 consecutive days. Experiments were performed in triplicate, untreated cells being used as positive controls. Results were expressed as percent of cell viability compared to untreated cells as Day 1.

***Patient-derived breast cancer xenograft***

One sub-cutaneous patient-derived xenograft of human HER2-overexpressing breast cancer was used in this study. It had been obtained from a skin metastasis biopsy before any medical treatment in a patient with initial sensitivity to trastuzumab-based therapy.

Nude mice, purchased from Janvier (Centre-Elevage-Janvier, France), were maintained in specific pathogen-free animal housing (SMBH, Bobigny, agreement n°C9300801). After the tumor biopsy had been performed, one sample was transported in RPMI-1640 and subcutaneously grafted in 6-week-old NMRI-nude mice, under xylasin (10mg/kg)/ketamin (100 mg/kg) anaesthesia. The Ministry of Research and Ethics Committee for experimental animal studies approved this study (APAFIS#17190-2018101814245111).

After a successful engraftment of the metastatic sample, a clinical score was recorded daily for the mice and tumor growth was measured in two perpendicular diameters with a caliper. Tumor volumes were calculated as $V=L\times l^{2}\div2$, L being the larger diameter (length), l the smaller (width). After mouse euthanasia by cervical dissociation, the tumor was resected, cut into small pieces of 1 mm^3^, and grafted again in 30 nude mice. The day when tumors reached a volume of 300 mm^3^ – i.e. 100% tumor volume – was considered as Day 0. Mice were divided into four groups: one group of untreated mice (N=10), and three groups treated over 21 days with intra-veinous injections of trastuzumab, Fab anti-HER2#1 fragment and Fab anti-HER2#2 fragment, at 1 mg/kg once a week (N=10 in each group). A daily clinical score was recorded and tumor growth measured weekly until tumor weight reached the ethically recommended limit of less than 10% of mouse weight (Directive 2010/63/EU of the European Parliament and the Council of 22 September 2010 on the protection of animals used for scientific purposes; Official Journal of the European Union L 276/33).

Ultrasonography was performed twice a week on treated and untreated mice with an AplioXT device (Toshiba, Japan) to assess tumor response.

We assumed a response rate of 80% with trastuzumab (8) with an unacceptable response rate of 50%, and that Fab antibodies will have at least the same anti-tumor efficacy than trastuzumab for a beta risk evaluation. We thus calculated a sample size from control of 20 mice needed to highlight a significant difference between control and trastuzumab, and an absence of difference between trastuzumab and the Fab. An intermediate analysis was done with 10 mice in each group. We performed a multiway analysis of variance (MANOVA) of tumor volume considering interaction between group of treatment (i.e. control, anti-HER2Fab#1, anti-HER2Fab#2 and trastuzumab) and the times of measures (between Day 9 and Day 21).

All *in vivo* experiments followed the ARRIVE guidelines for animal research (9).

At the time of euthanasia, for each mouse, the tumours and the different organs (liver, spleen, kidneys, ovaries, brain and lungs) were systematically analysed. Tumours were dissected and divided into three parts: one part was immediately snap-frozen in liquid nitrogen, one part was formalin-fixed (fixing agent AFA, CARLO ERBA Reagents) and paraffin-embedded, one part was glutaraldehyde-fixed and Epon resin-embedded.

Before each intravenous injection, a blood sample was taken for pharmacokinetic analysis.

***Tissue effects of anti-HER2 Fab fragment and trastuzumab***

Necrosis areas were evaluated on H&E colored 2 µm-thick paraffin sections. When present, necrosis was delineated on virtual slides created on a Nanozoomer2.0H scanner (Hamamatsu/ Japan), and quantified using DotSlide2 software. Results were expressed as the sum of necrotic areas for each section, and the mean ± SEM. Anti-proliferative effect was observed by Ki67 immunostaining in 5 µm-thick sections, on 5 different non necrotic fields at X200 magnifications, and quantified by percentage of positive cells/HPF (high power field). Anti-angiogenic effect was assessed using CD31 immunostaining in 5 µm-thick sections, on 5 different non necrotic fields at X200 magnifications, and quantified by number of positive endothelial cells/HPF. Pro apoptotic effect was assessed by cleaved-caspase 3 immunostaining in 5 µm-thick sections, on 5 different non necrotic fields at 200 magnifications, and quantified by percentage of positive cells/HPF. Staining was performed using an indirect immunoperoxydase method with a rabbit anti-Human Ki67 antibody (dilution 1:100, abcam), a rat anti-Mouse CD31 antibody (dilution 1:20, Dianova), and a rabbit anti-Human Cleaved caspase-3 antibody (dilution 1:50, Cell Signaling Technology) as primary antibodies.

All tissue sections were observed under an Olympus AX 70 microscope and analyzed using

CellSens Dimension software (Olympus).

***Toxicity analysis***

Ten µm-thick heart sections from snap frozen samples of mice hearts were prepared in RLT buffer (Qiagen) and RNA was extracted using Qiagen RNeasy mini kit. RNA quality was assessed by spectrometric assay (NanoDrop® ND-1000, Thermo scientific). Fifty ng/mL of RNA was extracted from samples.

Quantification of mRNA expression of adrenomedullin and BNP was performed with RT-qPCR assay using GoScript™-Reverse-Transcription System (Promega, France), GoTaq® qPCR Master Mix (Promega, France), and taqman primers and probes for *Adrenomedullin* (mM00437438_G1, Thermofisher) and *BNP* (mM01255770_g1, Thermofisher). A total mix volume of 120 µL for retrotranscription and 324 µL per probe for RT-qPCR was used. Assays were read at 95°C for 60 cycles on the Biorad Real-Time Detection System. TBP (Hs99999910_m1) (Life-Technologies) and GAPDH (Hs02786624_g1) (ThermoFisher Scientific) was used as the endogenous control for normalization. Data were normalized on the reference gene, using CFX manager software and expression levels were calculated using the 2^-ΔCq^ method. All Cq value >40 was not retained for analysis.

Each RT-qPCR assay was performed according to the MIQE guidelines (minimum information for publication of quantitative real-time PCR experiment) and conducted in triplicate (10).

For western blot analysis of DPP3 and cleaved-caspase 3, 7 µm-thick heart sections from snap frozen samples of mice hearts were prepared in RIPA lysis buffer (50 mM Tris HCl pH7.4, 1 mM EDTA, 150 mM NaCl) in the presence of anti-proteases and anti-phosphatases (Sigma-Aldrich, St. Louis, USA). After centrifugation (12,000 RPM, 10 min, 4°C), protein concentration was measured by spectrophotometry using the Pierce BCA Protein Assay kit (ThermoFischer Scientific, France).

Samples were diluted in the extraction buffer (20 μg) and loading buffer then heated at 99 °C for 7 min for protein denaturation. Proteins were separated by electrophoresis on SDS-page 4-20% polyacrylamide gel (Biorad, Hercules, USA), transferred to a nitrocellulose membrane (0.45 μm) and stained with ponceau red. The non-specific sites of the membranes were saturated with a mixture of TBST and 5% milk. Membranes were incubated with the following primary antibodies: humanized anti-DPP3 antibody (1/1,000, 4TEEN4, Berlin, Germany), and cleaved caspase-3 (Asp175) antibody (Cell Signaling) overnight at 4°C. Blots were then incubated with rabbit anti-human IgG (1/10,000; Thermo Fisher Scientific, Watham, USA) coupled with peroxidase 1 hour at 21°C. Peroxidase activity was subsequently revealed with ECL Prime (GE Healthcare). GAPDH was taken as a charge control. Chemiluminescence was detected using LAS 3000 (Fuji) and measured using MultiGauge V2.02 software (Fuji).

**Relevance of the HER2 overexpressing xenograft model**

Immunochemistry was performed on tumor xenografts 5 μm-thick paraffin sections with an indirect immunoperoxydase method using rabbit anti-Human HER2 (dilution 1:100, cloneSP3, Spring Bioscience) as the primary monoclonal antibody. Tissue sections were analyzed under an Olympus AX 70 microscope at X400 magnification.

We assessed *HER2* copy number on DNA extracted from tumors using the QIAamp® DNA Mini-Kit (Qiagen). DNA quality was assessed by spectrometric assay (NanoDrop® ND-1000, Thermo scientific). Each droplet digital PCR assay was performed according to the MIQE guidelines and conducted in triplicate. Reagent mixes (with Hs00223586_cn ERBB2 as the primer and TaqMan® Copy Number Reference Assay, human, RNase P, Life Technologies) were prepared using standard Taqman primer/probe chemistry with a 2 X ddPCR Mastermix (BioRad, Laboratories), a 20 X primer/probe (900/250 nM), and 5 μL of sample DNA template in a final volume of 20 μL. The reagent mixture was loaded into an eight-channel droplet generator (BioRad, Laboratories). Seventy μL of droplet generation oil were loaded for each channel and after generation of water-in-oil droplets the droplets were transferred to a 96-well PCR plate and placed in a Biorad thermocycler. An initial denaturation step (95 °C, 10 min) was followed by 45 cycles at 95 °C for 15 s and at 60 °C for 1 min. The PCR products were streamed through a droplet reader and the results were analyzed using QuantaSoft software (BioRad Laboratories). All droplets were gated on the basis of detector peak width to exclude doublets or triplets.

**Pharmacokinetic study of anti-HER2 antibodies after intraventricular administration**

***ELISA procedures for anti-HER2 Fab fragment and trastuzumab concentration assessment in serum, brain and cerebrospinal fluid***

For Fab anti-HER2 concentration assessment, we used the same methodology than described above for ranibizumab. Plates were coated with 0.25 mg/L of HER2 (sigma Aldrich, USA). Anti-human kappa light chain (Sigma) coupled with peroxidase, diluted in 1% PBS-BSA were used as secondary antibody.

Trastuzumab, bevacizumab and ranibizumab were provided by the pharmacy of Avicenne Hospital. For trastuzumab concentration assessment, we used an ELISA procedure already validated for clinical practice (11).

***Blood and CSF samples***

For this step we aim to quantify the pharmacokinetic differences between the anti-HER2 Fab fragment and IgG trastuzumab. For the first analysis, 36.5 µg of trastuzumab and anti-HER2 Fab were prepared in saline solution (0.9%) in a total volume of 100 µL. In the second analysis, 1400 µg of trastuzumab and anti-HER2 Fab were prepared in saline solution in a total volume of 100 µL.

We injected a total volume of 100 µL of each solution into the catheter to the cisterna magna of 17 rats. Then 50 µL of cerebrospinal fluid and blood was sampled at 0 (before the injection of the solution), 30 minutes, 1 h, 4 h and until 1 week after the CSF injection of antibodies. Blood was centrifugated at 10,000 RPM for 10 minutes to isolate serum. Cerebrospinal fluid and serum were then analyzed with an in-house ELISA method (described above).

***Analysis of brains***

We assessed quantitative parenchymal penetration of antibodies on different functional parts of the brain (cortex, cerebellum and central area) at two time points. A first series of brains was analyzed at an early time point of 240 minutes after intrathecal administration, a second series was analyzed at the end of the procedure, 7 days after intrathecal administration. Rats were deeply anesthetized and euthanatized by exsanguination and brain were removed and processed for tissue analysis. Brains were macroscopically divided in 3 longitudinal sections: The central section was formalin-fixed and paraffin-embedded (FFPE) for immunohistochemical analyses. One lateral part was frozen for immunohistochemical analyses, and the second lateral part was immediately divided into three distinct areas (cortical, central and posterior) and frozen for further ELISA assessment.

For antibody concentration assessment in brain, we used the same ELISA as described above. Each frozen part of the brain (cortical, central, posterior) was prepared using protein extraction agent (N-PER™ Neuronal Protein Extraction Reagent, Thermo Fisher Scientific, USA), with a ratio of 1 g tissue for 10 mL of N-PER reagent. Samples were homogenized in N-PER reagent for 10 minutes, and centrifugated at 10,000 RPM for 10 minutes at 4°C. Supernatant were collected for further ELISA analysis.

Appropriate controls were implemented, on rat brains which did not receive any antibody injection.

***Pharmacokinetic modeling***

The pharmacokinetic of trastuzumab and of the anti-HER2 Fab#2 in rats were assessed independently by a population approach using Monolix 2020R1 (Lixoft, Antony, France). A two-compartment model with two first-order elimination rates and an absorption compartment was implemented to describe the concentrations measured in serum and CSF. Proportional error models were used to describe residual variabilities in serum and CSF concentrations. Brain distribution of trastuzumab and anti-HER2 Fab#2 were assessed comparing typical values of parameters describing CSF-to-serum flow and elimination in CSF for each antibody.

**Statistical analysis**

For the comparison of the means, we used the Student T-test (2 means) for series distributed according to the normal distribution. To compare quantitative variables from multiple samples in the pharmacokinetic analysis, we used a multiparametric ANOVA test. All statistical analyses were performed using R software.

**References**

1. Latvala S, Jacobsen B, Otteneder MB, Herrmann A, Kronenberg S. Distribution of FcRn Across Species and Tissues. J Histochem Cytochem. 2017;65(6):321-33.

2. Huang X, Zheng F, Zhan CG. Binding structures and energies of the human neonatal Fc receptor with human Fc and its mutants by molecular modeling and dynamics simulations. Mol Biosyst. 2013;9(12):3047-58.

3. Ternant D, Ceze N, Lecomte T, Degenne D, Duveau AC, Watier H, et al. An enzyme-linked immunosorbent assay to study bevacizumab pharmacokinetics. Ther Drug Monit. 2010;32(5):647-52.

4. Bonneau C, Paintaud G, Tredan O, Dubot C, Desvignes C, Dieras V, et al. Phase I feasibility study for intrathecal administration of trastuzumab in patients with HER2 positive breast carcinomatous meningitis. Eur J Cancer. 2018;95:75-84.

5. Bousquet G, Darrouzain F, de Bazelaire C, Ternant D, Barranger E, Winterman S, et al. Intrathecal Trastuzumab Halts Progression of CNS Metastases in Breast Cancer. J Clin Oncol. 2016;34(16):e151-5.

6. Nguyen TT, Angeli E, Darrouzain F, Nguyen QT, Desvignes C, Rigal M, et al. A successful compartmental approach for the treatment of breast cancer brain metastases. Cancer Chemother Pharmacol. 2019;83(3):573-80.

7. Durand M, Oger M, Nikovics K, Venant J, Guillope AC, Jouve E, et al. Influence of the Immune Microenvironment Provided by Implanted Biomaterials on the Biological Properties of Masquelet-Induced Membranes in Rats: Metakaolin as an Alternative Spacer. Biomedicines. 2022;10(12).

8. Baselga J, Cortes J, Kim SB, Im SA, Hegg R, Im YH, et al. Pertuzumab plus trastuzumab plus docetaxel for metastatic breast cancer. N Engl J Med. 2012;366(2):109-19.

9. Percie du Sert N, Hurst V, Ahluwalia A, Alam S, Avey MT, Baker M, et al. The ARRIVE guidelines 2.0: Updated guidelines for reporting animal research. PLoS Biol. 2020;18(7):e3000410.

10. Bustin SA, Benes V, Garson JA, Hellemans J, Huggett J, Kubista M, et al. The MIQE guidelines: minimum information for publication of quantitative real-time PCR experiments. Clinical chemistry. 2009;55(4):611-22.

11. Bernadou G, Campone M, Merlin JL, Gouilleux-Gruart V, Bachelot T, Lokiec F, et al. Influence of tumour burden on trastuzumab pharmacokinetics in HER2 positive non-metastatic breast cancer. Br J Clin Pharmacol. 2016;81(5):941-8.
