# Supplementary material for: A Fab of trastuzumab to treat HER2 overexpressing breast cancer brain metastases
Source: Exp Hematol Oncol. 2024 Apr 15;13:41. doi: 10.1186/s40164-024-00513-7 (PMC11017592; doi:10.1186/s40164-024-00513-7)
Supplement: Supplementary file 13 — Supplementary Material 13 [file 40164_2024_513_MOESM13_ESM.docx]

**A Fab of trastuzumab to treat HER2 overexpressing breast cancer brain metastases**

Running title: a Fab of trastuzumab to treat brain metastases

Eurydice Angeli^1,2,3,&^, Justine Paris^1,&^, Olivier Le Tilly^4,5,6^ , Céline Desvignes^4,5^, Guillaume Gapihan^1^, Didier Boquet^7^, Frédéric Pamoukdjian^1,2,3^, Diaddin Hamdan^1^, Marthe Rigal^8^, Florence Poirier^3,9^, Didier Lutomski^3,9^, Feriel Azibani^1^ Alexandre Mebazaa^1,10^, Amaury Herbet^7^, Aloïse Mabondzo^7^, Géraldine Falgarone^1,3,11^, Anne Janin^1*^, Gilles Paintaud^4,5,6,*^, Guilhem Bousquet^1,2,3,*^

^1^ Université Paris Cité, INSERM, UMR_S942 MASCOT, F-75006, Paris, France.

^2^ APHP, Hôpital Avicenne, Department of medical oncology, F-93000, Bobigny, France.

^3^ Université Sorbonne Paris Nord, 99 Avenue Jean Baptiste Clément, F-93430 Villetaneuse, France.

^4^ Université de Tours, INSERM, U1327 ISCHEMIA EA4245, Tours, France.

^5^ CHRU de Tours, Centre Pilote de suivi Biologique des traitements par Anticorps (CePiBAc), Tours, France.

^6^ Tours University Hospital, Pharmacology Department, Tours, France.

^7^ Université Paris-Saclay, CEA, DMTS, SPI, LENIT, F-91191 Gif-sur-Yvette, France.

^8^ APHP, Hôpital Avicenne, Department of Pharmacy, F-93000 Bobigny, France.

^9^ Unité de Recherche en Ingénierie Tissulaire-URIT, Sorbonne Paris Nord University, 99 Avenue Jean Baptiste Clément, F-93430 Villetaneuse, France.

^10^ APHP, Hôpital Lariboisière, Department of Anesthesia and Critical Care, F-75010 Paris, France.

^11^ APHP, Hôpital Avicenne, Unité de Médecine Ambulatoire, F-93009 Bobigny, France.

^&^These authors are co-first authors, *These authors are co-senior authors

Corresponding authors: Pr. Guilhem Bousquet, Service d’Oncologie Médicale, AP-HP Hôpital Avicenne, Bobigny F-93000, France. E-mail: [guilhem.bousquet@aphp.fr](mailto:guilhem.bousquet@aphp.fr), Tel +33(0)1.48.38.85.29 ; Dr Eurydice Angeli, Service d’Oncologie Médicale, AP-HP Hôpital Avicenne, Bobigny F-93000, France. E-mail: eurydice.angeli@gmail.com, Tel +33(0)1.48.38.85.29

**Abstract**

***Background***

Despite major therapeutic advances for two decades, including the most recently approved anti-HER2 drugs, brain metastatic localizations remain the major cause of death for women with metastatic HER2 breast cancer. The main reason is the limited drug passage of the blood-brain barrier after intravenous injection and the significant efflux of drugs, including monoclocal antibodies, after administration into the cerebrospinal fluid. We hypothesized that this efflux was linked to the presence of a FcRn receptor in the blood-brain barrier.

***Methods***

To overcome the brain-to-blood efflux, we engineered two Fab fragments of trastuzumab, an anti-HER2 monoclonal antibody, and did a thorough preclinical development for therapeutic translational purpose, except toxicity studies on bigger animals. For the pharmacokinetic studies of intra-cerebrospinal fluid administration, we implemented original rat models with catheter implanted into the cisterna magna.

***Results***

We demonstrated the safety and equal efficacy of the Fabs with trastuzumab in vitro, and in vivo using a patient-derived xenograft model of HER2 overexpressing breast cancer. After intraventricular administration in rats, we demonstrated that the brain-to-blood efflux of Fab was up to 10 times lower than for trastuzumab, associated with a two-fold higher brain penetration compared to trastuzumab.

***Conclusion***

We successfully engineered and thoroughly conducted the preclinical development of a trastuzumab Fab as effective as the native monoclonal antibody, and capable of significantly reducing brain-to-blood efflux and doubling brain penetration after intra-cerebrospinal fluid injection. This Fab could thus be a new and original effective drug in the treatment of HER2 breast cancer brain metastases, which will be demonstrated by a phase I clinical trial dedicated to women in resort situations.

**Keywords:**HER2 breast cancer, brain metastases, Fab, trastuzumab, pharmacokinetic.

**Background**

Brain metastases occur in 20% of cancers (1) and are associated with a median survival less than 6 months, challenging daily practice in oncology (2). In women with metastatic HER2 breast cancer, combined anti-HER2 treatments with trastuzumab and pertuzumab, two monoclonal IgG antibodies, lead to chemo-curability with 37% of long survivors (3, 4). In contrast, up to 50% of women will develop brain metastases and die because of these metastatic localizations, despite standard medical treatments that include surgery and radiation therapy (5, 6). Incidence of brain metastases has increased in the last ten years, as a result of a better control of localizations outside the central nervous system, but also because most anti-cancer drugs are unable to cross the blood-brain-barrier (BBB) at pharmacologically relevant concentrations [reviewed in (7)]. Two recently approved anti-HER2 drugs, tucatinib and trastuzumab-deruxtecan, have benefited to the treatment of brain metastases. Tucatinib is a tyrosine kinase inhibitor blocking the intracellular part of HER2 signaling pathway, with a better BBB diffusion compared to other anti-HER2 tyrosine kinase inhibitors (8), with promising cerebral responses of 40% in heavily pretreated patients (9, 10). Trastuzumab-deruxtecan, an antibody-drug conjugate, has led to a 73% rate of intracranial responses, with 27% of complete cerebral responses (11, 12). However, despite these promising initial results, both drugs have a time-limited efficacy (10-12)^,^(13), with no durable complete response and a median progression-free survival up to 18 months, justifying to identify new therapeutic approaches in these resort situations.

Several approaches are being explored to improve drug penetration into the brain, which comprise the use of bi-specific antibodies, intranasal way of administration or mechanical disruption of the BBB [reviewed in (7)]. To avoid the limited blood-to-brain passage of the BBB, a direct administration of drugs into the cerebrospinal fluid (CSF) has been proposed in the treatment of meningeal carcinomatosis (14-16). In a pilot pharmacological study, we have demonstrated the benefit of using this compartmental approach to treat parenchymal brain metastases in a young woman with progressive HER2-overexpressing breast cancer. To reach relevant concentrations of trastuzumab in the CSF, repeated intraventricular injections three times a week were required because of a major drug efflux from brain to blood. However, such a difficult and risky procedure cannot be generalizable for daily care (17, 18).

According to preclinical data in murine models, this efflux of IgG was saturable by Fc but not by F(ab’)2 fragments (19). Despite the physiological brain distribution and clearance of most molecules, driven by pressure gradients along arterial and venous perivascular spaces through a system called the glymphatic system (20), there are drug transporters responsible for an active efflux of xenobiotics from brain to blood (19, 21, 22).

We hypothesized that an Fc receptor (FcRn) expressed by endothelial cells from the BBB is responsible for this efflux of therapeutic IgG and thus of trastuzumab. To avoid the brain-to-blood efflux, we engineered a fragment antigen-binding (Fab) of trastuzumab and characterized it in preclinical models for translational therapeutic purpose.

**Methods**

**FcRn expression in rat brain**

Brains from five untreated *Rattus norvegicus* (Charles River, France) aged of 6 weeks were carefully removed after euthanasia, formalin-fixed, paraffin-embedded and cut into 5 µm-thick sections. FcRn expression was assessed with an indirect immunoperoxydase method, using a goat anti-rat FcRn as the primary antibody (R&D Systems; AF6775), and a biotinylated rabbit anti-goat IgG as the secondary antibody (Vector Laboratories, Inc., Burlingame, CA). Standardized controls were the absence of primary antibody and the use of an irrelevant primary antibody of the same isotype. Tissue sections were analyzed under an Olympus AX 70 microscope with a 0.344-mm^2^ field size at X400 magnification. Analyses were performed by two pathologists (GB, AJ).

**Proof of concept with anti-VEGF antibodies**

To validate the absence of efflux of Fab fragments, we first used two commercialized anti-VEGF antibodies, bevacizumab (full-length IgG, 149 kDa) and ranibizumab (Fab, 48 kDa) for a proof of concept.

***Enzyme-linked Immunosorbent Assay (ELISA) procedures for bevacizumab and ranibizumab concentration assessment in serum, brain and cerebrospinal fluid***

Bevacizumab and ranibizumab were provided by the pharmacy of Avicenne Hospital. For ranibizumab concentration assessment, we developed a protocol based on the procedure implemented by Ternant *et al* to assess bevacizumab serum concentrations in patients (23). Microtiter 96-well plates were prepared by incubating 100 $\mu$L VEGF_165_ at a concentration of 0.25 mg/L in the coating buffer (1 mol/L carbonate–bicarbonate buffer) overnight at 4°C. The plates were washed four times with PBS containing 0.05% Tween 20. The remaining protein-binding sites were blocked by 2 hours incubation at room temperature with 200 $\mu$L blocking buffer (PBS-BSA 1%). Plates were washed 4 times and 100 $\mu$L of 1:100 diluted standards, quality controls (QCs), and samples were added. After incubating the plates for 2 hours at 37°C and following a new washing round, 100 $\mu$L of anti-human secondary antibodies (anti-human kappa light chain, Sigma 1:3500 diluted) coupled with peroxidase, diluted in 1% PBS-BSA was added to each well. After 1 hour at room temperature followed by washing, 100 $\mu$L OPD (prepared by dissolving tablet sets in 20 mL distilled water) was added at room temperature in the dark. The color reaction was stopped after 10 minutes by adding 50 $\mu$L of 2 mol/L sulfuric acid per well. Reading was performed at two wavelengths (490 and 630 nm) using an ELISA plate reader (CLARIOstare, BMG Labtech). The absorbance at 620 nm corresponds to the background signal linked to the plate and was subtracted from the absorbance at 492 nm.

For the accuracy of quality control and standard curve determination, we used values with the better inter-day accuracy, determined as coefficient of variation <20% (reflect of the reproducibility) and bias from expected concentrations <20%. After their determination, each quality control and standard points were tested again 12 times to ensure that coefficient of variation was <20% and bias from expected concentrations was <20% (validation step). The limit of detection was calculated by assaying 12 replicates of blank samples at 3-times of the mean standard deviations. The lower limit of quantification corresponded to the lowest calibration standard. The upper limit of quantification was defined as the highest amount of ranibizumab that could be quantified with standard deviation and relative error *<*20%. To ensure that samples can be diluted in case of peak concentrations outside of the standard curve, we tested the reproducibility of a series of increasing dilutions, corresponding to dilution linearity.

For bevacizumab we used an ELISA procedure already validated for clinical practice (14, 17, 18, 23). We used an anti-human IgG Fc specific as secondary antibody, coupled with peroxidase (Sigma).

***Implantation of catheter into the cisterna magna of rats, preparation of samples***

We developed our own method of cisterna magna catheterization across the occipital crest in *Rattus norvegicus* (Wistar, Charles River, France) aged of 6 weeks. During the whole experiment, rats were maintained in environment adapted to their housing conditions. The use of rats as well as catheterization of the cisterna magna has been approved by the Ministry of Research (APAFIS#17189-2018101814041264), and by the Ethics Committee on animal experiments.

We used a special surgical setup for minimally invasive repeated sampling and injections that can be used for a prolonged time.

The rat was anesthetized with isofluorane 3% and a mixture of buprenorphine at 0.3 mg/mL (0.05 mg/kg), ketamine at 100 mg/mL (90 mg/kg), xylazine at 100 mg/mL (10 mg/kg). It is then placed on a stereotactic frame with the head positioned in order to achieve a flat skull. The operating area is shaved and cleaned using dermic betadine. Skin is incised on 2-3 cm length from the dorsal midline of the skull to the occipital crest. Using blunt scissor, skin is disjoined from the skull. The skull is then exposed by gently scraping the periosteum with a scalpel. Using the stereotactic frame, a 0.7 mm hole is drilled into the interparietal bone, 1 millimeter ahead of the occipital crest on the sagittal midline, at an angle of 60-70° from the horizontal plan, in the caudal direction, at 5 mm depth. Catheter (SAI infusion Technologies) is then placed into the hole and fixed at the skull with a drop of tissue glue (histoacryl, Braun). Cutaneous plan is then sutured, and the end of the catheter is placed under the skin.

To analyze the intrathecal pharmacokinetics after intrathecal injection of ranibizumab or bevacizumab, two solutions were prepared. Fourteen μg of bevacizumab and ranibizumab solutions were diluted in saline (0.9%) to achieve a total volume of 100 µL. Each solution was injected into the cisterna magna using the catheter, in a total of 10 rats (5 rats per group). Then, 50 μL of cerebrospinal fluid and blood were taken at 0 (before injection of the solution), 30 minutes, 60 minutes, 180 minutes and 240 minutes after injection. Once collected, the blood tube was centrifuged at 10,000 RPM at 4°C for 10 minutes to isolate the serum. The cerebrospinal fluid and serum were then analyzed with the developed ELISA technique.

**Engineering of anti-HER2 Fab fragments**

Two anti-HER2 Fab fragments were engineered for this study, using the same methodological approach: one in our research team, and the second in collaboration with BIOTEM®.

For the Fab fragment we have engineered (Fab#1), light chain and heavy chain of the Fab were synthesized separately and then assembled into chinese hamster ovary (CHO) cells. The light chain was synthesized in pcDNA3.2 vector, using the sequence corresponding to the light chain of trastuzumab and the heavy chain was synthesized in pFUSEss-CHIg-hG1 vector (Invitrogen, USA). A stop codon was added at the end of each sequence to avoid adding other elements to the sequence of interest.

Sub-cloning in expression vector was realized in *E.Coli XL1-Blue bacteria*. *E.Coli* were first transformed with genes or vectors separately by heat shock at 42°C for 30 seconds followed by 2 minutes on ice, to amplify them. Bacteria were then incubated in a rich SOC medium (Invitrogen, USA) for 1 hour at 37°C at 230 RPM and then cultured in a liquid environment containing the appropriate selection antibiotic. The tubes were incubated at 37°C overnight. For each gene or vectors, plasmid DNA was recovered with the Wizard Plus SV Minipreps DNA Purification System Kit (Promega, USA), which allows the production of a clarified lysat and the purification of plasmid DNA. After a PCR with the purified plasmids, the products were digested with Dpn1 (New England BioLabs, France) for 2 hours in order to get rid of bacterial DNA. Products were purified again before being controlled on 1% agarose gel (Invitrogen, USA).

Once the size of genes and vectors was controlled by gel migration, vectors and genes were treated 2 hours with T4-DNA polymerase (New England BioLabs, France) to generate blunt ends. *E.Coli* were transformed by heat shock with the gene of the Fab light chain or the heavy chain, their vector and its specific primers. Each gene and its corresponding vectors were assembled with specific primer. A colony-based PCR was performed. Each colony collected was amplified and PCR products were controlled on 2% agarose gel (Invitrogen, USA). For each product the plasmid DNA was recovered, sequenced, and analyzed by alignment with the expected sequence.

Insertion in synthesis vector (CHO) was performed to synthesize the Fab. The CHO cells were seeded 24 hours before transfection with 70 to 90% confluence in 6 wells plates. The two plasmids (the light chain and heavy chain) were placed together in the culture medium, with a transfection agent (Sigma-Aldrich) at a 3:1 ratio during 15 minutes at 20°C before being added to each well. After 24 hours, the antibiotics were added to achieve 50% mortality of the cells and then gradually increased to 100% mortality of non-transfected cells. After several days, the culture medium was removed and purified by column chromatography or concentrated (Vivaspin Turbo 15R, Sartorius). A SDS-PAGE gel was then performed to ensure the weight of the Fab. A Western Blot was performed with anti-trastuzumab antibody (R&D SYSTEM). To control the amino-acid sequence of the Fab fragments by tandem mass spectrometry (MS/MS), the protein bands corresponding to the to the light and heavy chains of the Fab were excised from the SDS-PAGE under reducing conditions and proteins were in-gel digested as previously described (24). Peptide samples were analyzed with a QTOF mass spectrometer (Impact HD, Bruker) equipped with the CaptiveSpray ion source (Bruker). The QTOF was coupled to a nano liquid chromatography (Ultimate 3000, ThermoFisher Scientific) running with two buffers: 0.1% formic acid in water and 0.1% formic acid in 80% acetonitrile. Chromatographic separation was carried out on a C18 reverse phase column (75 μm, 150 mm, 120 Å Wide Pore, Bruker) with a gradient elution at a flow rate of 300 nL/min during 60 min. The system was operated with automatic switching between MS and MS/MS modes using a data-dependent acquisition (DDA) method for peptide fragmentation. MS/MS spectra were then processed with the Data Analysis software (Bruker). In a first step, peptides have been identified automatically using the MASCOT software (Matrix Science, London, UK) and the SwissProt database (www. expasy.org) with the following parameters: carbamidomethylation for cysteine residues; potential oxidation for methionine residues; tolerance on mass measurements of 20 ppm in MS mode and 0.1 Da in MS/MS mode; enzymatic cleavage by trypsin with one missed cleavages allowed. The species of origin was restricted to the Mammalia. In a second step, peptides not automatically identified, were then manually sequenced on the basis of their MS/MS spectra using the annotate tool in the Data Analysis software (Bruker).

For the Fab fragment synthesized in collaboration with BIOTEM® (anti-HER2Fab#2), the synthetic genes encoding the anti-HER2 Fab light and heavy chains were designed and controlled by sequencing, then subcloned in BIOTEM® proprietary vector and transfected in CHO cells. The supernatant was then purified by affinity chromatography and controlled by size exclusion chromatography.

**Validation of anti-tumoral effect of Fab anti-HER2 fragments compared to trastuzumab**

***Human cancer cell lines***

Two breast cancer cell lines were used, BT474 with HER2 overexpression and MDA-MB-231 which does not overexpress HER2. The cell lines were obtained from ATCC. These cells were cultured at 37 °C under normoxic conditions (20 % of O_2_ and 5 % of CO_2_) in RPMI-1640 medium supplemented with 10 % of fetal calf serum and 1 % antibiotics.

***In vitro affinity and cytotoxicity of anti-HER2 Fab antibodies***

The ability of trastuzumab or Fab anti-HER2 fragments to efficiently bind to HER2 membrane receptors was assessed on the BT-474 and MDA-MB-231 cell lines. The two cell lines were grown separately on culture slides (BD Falcon™). Five micrograms of commercial trastuzumab (Roche) or anti-HER2 Fab fragments coupled with Alexa Fluor 488 fluorophore (using APEX™ Alexa Fluor™ 488 Antibody Labeling Kit, Invitrogen) were incubated for 1 hour with each type of human cancer cell line. Then, the cells were fixed in acetone at 4°C, the nuclei were stained with DAPI (Vector Laboratories, Vectashield, H-1200) and fluorescence staining was observed at X400 magnification. The experiment was conducted five times independently, and a minimum of 100 cells were analysed.

For saturation binding experiment, BT474 cells were cultured in RPMI1640 supplemented with 10% fetal calf serum, 2 mM L-glutamine, 100 U/mL penicillin and 100 µg/mL streptomycin. Cells were maintained at 37°C in a humidified atmosphere of 5% CO_2_. All media and cell culture supplements were from Life Technologies. Collected cells were seeded (300,000 cells/tube). Cells were incubated overnight at 4°C with 600 µL of D-phosphate-buffered saline (PBS) supplemented with 0.1% BSA and 5% normal goat serum (NGS, Life Technologies) and containing increasing concentrations of trastuzumab or anti-HER2Fab antibodies. After two washes with 1000 µL of ice-cold D-PBS, and in order to reveal the binding, cells were incubated for 4h at 4°C in the dark with Goat anti-Human Kappa Light Chain Secondary Antibody, FITC (Invitrogen catalog #A18854) in 300 µL of D-PBS supplemented with 0.1% BSA and 5% normal goat serum. After two washes with 1000 µL of ice-cold D-PBS, fluorescence was measured using a FACSCalibur (BD BIOSCIENCES). Then mean fluorescence intensity (MFI) of samples was then determined by cytometer software. The MFI results were analyzed and curves were fitted using GraphPad Prism with the adequate dose-response and IC50±0.1 SD values were determined.

To assess anti-HER2 Fab fragment cytotoxicity, BT474 cells were seeded in 96-well tissue culture plates at a density of 5.10^3^ cells per well. After 24 hours of incubation, the cells were exposed to increasing concentrations of anti-HER2 Fab fragment or trastuzumab (0 to 8 µg/mL) for 72 additional hours. Cell viability was determined by the colorimetric conversion of yellow, water-soluble tetrazolium MTT (3-[4, 5-dimethylthiazol-2-yl]-2,5-diphenyl-tetrazolium-bromide; Sigma), to purple, water-insoluble formazan. After incubation for 4 h at 37 °C with 0.4 mg/mL of MTT, the cells were placed in 0.1 mL of DMSO, and the absorbance was measured at 560 nm using a CLARIOstar® plate reader (BMG LABTECH). Experiments were performed in triplicate, untreated cells being used as positive controls, and drug-containing medium without cells as a negative control. Results were expressed as percent of cell viability compared to untreated cells.

To assess anti-HER2 Fab fragment inhibition proliferation effect, BT474 cells were seeded in 96-well tissue culture plates at a density of 5.10^3^ cells per well. Then, 8 µg/mL of anti-HER2 Fab or trastuzumab were added and cells were counted each day for 5 consecutive days. Experiments were performed in triplicate, untreated cells being used as positive controls. Results were expressed as percent of cell viability compared to untreated cells as Day 1.

***Patient-derived breast cancer xenograft***

One sub-cutaneous patient-derived xenograft of human HER2-overexpressing breast cancer was used in this study. It had been obtained from a skin metastasis biopsy before any medical treatment in a patient with initial sensitivity to trastuzumab-based therapy.

Nude mice, purchased from Janvier (Centre-Elevage-Janvier, France), were maintained in specific pathogen-free animal housing (SMBH, Bobigny, agreement n°C9300801). The tumor biopsy had been subcutaneously grafted in 6-week-old NMRI-nude mice, under xylasin (10mg/kg)/ketamin (100 mg/kg) anaesthesia. The Ministry of Research and Ethics Committee for experimental animal studies approved this study (APAFIS#17190-2018101814245111).

After a successful engraftment of the metastatic sample, a clinical score was recorded daily for the mice and tumor growth was measured in two perpendicular diameters with a caliper. Tumor volumes were calculated as $V=L\times l^{2}\div2$, L being the larger diameter (length), l the smaller (width). After mouse euthanasia by cervical dissociation, the tumor was resected, cut into small pieces of 1 mm^3^, and grafted again in 30 nude mice. The day when tumors reached a volume of 300 mm^3^ – i.e. 100% tumor volume – was considered as Day 0. Mice were divided into four groups: one group of untreated mice, and three groups treated over 21 days with intra-veinous injections of trastuzumab, Fab anti-HER2#1 fragment and Fab anti-HER2#2 fragment, at 1 mg/kg once a week (N=10 in each group). A daily clinical score was recorded and tumor growth measured weekly until tumor weight reached the ethically recommended limit of less than 10% of mouse weight (Directive 2010/63/EU of the European Parliament and the Council of 22 September 2010 on the protection of animals used for scientific purposes; Official Journal of the European Union L 276/33).Ultrasonography was performed twice a week on treated and untreated mice with an AplioXT device (Toshiba, Japan) to assess tumor response.

We assumed a response rate of 80% with trastuzumab (3) with an unacceptable response rate of 50%, and that Fab antibodies will have at least the same anti-tumor efficacy than trastuzumab for a beta risk evaluation. We thus calculated a sample size from control of 20 mice needed to highlight a significant difference between control and trastuzumab, and an absence of difference between trastuzumab and the Fab. An intermediate analysis was done with 10 mice in each group. We performed a multiway analysis of variance (MANOVA) of tumor volume considering interaction between group of treatment (i.e. control, anti-HER2Fab#1, anti-HER2Fab#2 and trastuzumab) and the times of measures (between Day 9 and Day 21).

All *in vivo* experiments followed the ARRIVE guidelines for animal research (25).

At the time of euthanasia, for each mouse, the tumours and the different organs (liver, spleen, kidneys, ovaries, brain and lungs) were systematically analysed. Tumours were dissected and divided into two parts: one part was immediately snap-frozen in liquid nitrogen and one part was formalin-fixed (fixing agent AFA, CARLO ERBA Reagents) and paraffin-embedded.

Before each intravenous injection, a blood sample was taken for pharmacokinetic analysis.

***Tissue effects of anti-HER2 Fab fragment and trastuzumab***

Necrosis areas were evaluated on H&E colored 2 µm-thick paraffin sections. Necrosis was delineated on virtual slides created on a Nanozoomer2.0H scanner (Hamamatsu/ Japan), and quantified using DotSlide2 software. Results were expressed as the sum of necrotic areas for each section, and the mean ± SEM. Anti-proliferative effect was observed by Ki67 immunostaining, anti-angiogenic effect was assessed using CD31 immunostaining and pro apoptotic effect was assessed by cleaved-caspase 3 immunostaining in 5 µm-thick sections, on 5 different non necrotic fields at X200 magnifications, and quantified by number of positive endothelial cells/HPF (high power field). Staining was performed using an indirect immunoperoxydase method with a rabbit anti-Human Ki67 antibody (dilution 1:100, abcam), a rat anti-Mouse CD31 antibody (dilution 1:20, Dianova), and a rabbit anti-Human Cleaved caspase-3 antibody (dilution 1:50, Cell Signaling Technology) as primary antibodies.

All tissue sections were observed under an Olympus AX 70 microscope and analyzed using

CellSens Dimension software (Olympus).

***Toxicity analysis***

Ten µm-thick heart sections from snap frozen samples of mice hearts were prepared in RLT buffer (Qiagen) and RNA was extracted using Qiagen RNeasy mini kit. RNA quality was assessed by spectrometric assay (NanoDrop® ND-1000, Thermo scientific).

Quantification of mRNA expression of adrenomedullin and BNP was performed with RT-qPCR assay using GoScript™-Reverse-Transcription System (Promega, France), GoTaq® qPCR Master Mix (Promega, France), and taqman primers and probes for *Adrenomedullin* (mM00437438_G1, Thermofisher) and *BNP* (mM01255770_g1, Thermofisher). A total mix volume of 120 µL for retrotranscription and 324 µL per probe for RT-qPCR was used. Assays were read at 95°C for 60 cycles on the Biorad Real-Time Detection System. TBP (Hs99999910_m1, Life-Technologies) and GAPDH (Hs02786624_g1, ThermoFisher Scientific) were used as the endogenous control for normalization. Data were normalized on the reference gene, using CFX manager software and expression levels were calculated using the 2^-ΔCq^ method. All Cq value >40 was not retained for analysis.

Each RT-qPCR assay was performed according to the MIQE guidelines and conducted in triplicate (26).

For western blot analysis of DPP3 and cleaved-caspase 3, 7 µm-thick heart sections from snap frozen samples of mice hearts were prepared in RIPA lysis buffer (50 mM Tris HCl pH7.4, 1 mM EDTA, 150 mM NaCl) in the presence of anti-proteases and anti-phosphatases (Sigma-Aldrich, St. Louis, USA). After centrifugation (12,000 RPM, 10 min, 4°C), protein concentration was measured by spectrophotometry using the Pierce BCA Protein Assay kit (ThermoFischer Scientific, France).

Samples were diluted in the extraction buffer (20 μg) and loading buffer then heated at 99 °C for 7 min for protein denaturation. Proteins were separated by electrophoresis on SDS-page 4-20% polyacrylamide gel (Biorad, Hercules, USA), transferred to a nitrocellulose membrane (0.45 μm) and stained with ponceau red. The non-specific sites of the membranes were saturated with a mixture of TBST and 5% milk. Membranes were incubated with the following primary antibodies: humanized anti-DPP3 antibody (1/1,000, 4TEEN4, Berlin, Germany), and cleaved caspase-3 (Asp175) antibody (Cell Signaling) overnight at 4°C. Blots were then incubated with rabbit anti-human IgG (1/10,000; Thermo Fisher Scientific, Watham, USA) coupled with peroxidase 1 hour at 21°C. Peroxidase activity was subsequently revealed with ECL Prime (GE Healthcare). GAPDH was taken as a charge control. Chemiluminescence was detected using LAS 3000 (Fuji) and measured using MultiGauge V2.02 software (Fuji).

**Relevance of the HER2 overexpressing xenograft model**

Immunochemistry was performed on tumor xenografts 5 μm-thick paraffin sections with an indirect immunoperoxydase method using rabbit anti-Human HER2 (dilution 1:100, cloneSP3, Spring Bioscience) as the primary monoclonal antibody. Tissue sections were analyzed under an Olympus AX 70 microscope at X400 magnification.

We assessed *HER2* copy number on DNA extracted from tumors using the QIAamp® DNA Mini-Kit (Qiagen). DNA quality was assessed by spectrometric assay (NanoDrop® ND-1000, Thermo scientific). Each droplet digital PCR assay was performed according to the MIQE guidelines and conducted in triplicate. Reagent mixes (with Hs00223586_cn ERBB2 as the primer and TaqMan® Copy Number Reference Assay, human, RNase P, Life Technologies) were prepared using standard Taqman primer/probe chemistry with a 2 X ddPCR Mastermix (BioRad, Laboratories), a 20 X primer/probe (900/250 nM), and 5 μL of sample DNA template in a final volume of 20 μL. Droplets were generated by a QX200 Droplet Generator (BioRad, Laboratories). PCR was carried out on the CFX96 Real Time System (Bio‐Rad).. An initial denaturation step (95 °C, 10 min) was followed by 45 cycles at 95 °C for 15 s and at 60 °C for 1 min. The PCR products were streamed through a droplet reader and the results were analyzed using QuantaSoft software (BioRad Laboratories). All droplets were gated on the basis of detector peak width to exclude doublets or triplets.

**Pharmacokinetic study of anti-HER2 antibodies after intraventricular administration**

***ELISA procedures for anti-HER2 Fab fragment and trastuzumab concentration assessment in serum, brain and cerebrospinal fluid***

For Fab anti-HER2 concentration assessment, we used the same methodology than described above for ranibizumab. Plates were coated with 0.25 mg/L of HER2 (sigma Aldrich, USA). Anti-human kappa light chain (Sigma) coupled with peroxidase, diluted in 1% PBS-BSA were used as secondary antibody.

Trastuzumab, bevacizumab and ranibizumab were provided by the pharmacy of Avicenne Hospital. For trastuzumab concentration assessment, we used an ELISA procedure already validated for clinical practice (27).

***Blood and CSF samples***

To quantify the pharmacokinetic differences between the anti-HER2 Fab fragment and IgG trastuzumab, 36.5 µg of trastuzumab and anti-HER2 Fab were prepared in saline solution (0.9%) in a total volume of 100 µL. For a second analysis, 1400 µg of trastuzumab and anti-HER2 Fab were prepared. We injected a total volume of 100 µL of each solution into the catheter to the cisterna magna of 17 rats. Then 50 µL of cerebrospinal fluid and blood was sampled at 0 (before the injection of the solution), 30 minutes, 1 h, 4 h and until 1 week after the CSF injection of antibodies. Blood was centrifugated at 10,000 RPM for 10 minutes to isolate serum. Cerebrospinal fluid and serum were then analyzed with an in-house ELISA method (described above).

***Analysis of brains***

We assessed quantitative parenchymal penetration of antibodies on different functional parts of the brain (cortex, cerebellum and central area) at two time points: 240 minutes and 7 days after intrathecal administration. Rats were euthanatized by exsanguination and brain were removed and processed for tissue analysis. Brains were macroscopically divided in 3 longitudinal sections: The central section was formalin-fixed and paraffin-embedded (FFPE) for immunohistochemical analyses. One lateral part was frozen for immunohistochemical analyses, and the second lateral part was immediately divided into three distinct areas (cortical, central and posterior) and frozen for further ELISA assessment.

For antibody concentration assessment in brain, we used the same ELISA as described above. Each frozen part of the brain was prepared using protein extraction agent (N-PER™ Neuronal Protein Extraction Reagent, Thermo Fisher Scientific, USA), with a ratio of 1 g tissue for 10 mL of N-PER reagent. Samples were homogenized in N-PER reagent for 10 minutes, and centrifugated at 10,000 RPM for 10 minutes at 4°C. Supernatant were collected for further ELISA analysis.

Appropriate controls were implemented, on rat brains which did not receive any antibody injection.

***Pharmacokinetic modeling***

The pharmacokinetic of trastuzumab and of the anti-HER2 Fab#2 in rats were assessed independently by a population approach using Monolix 2020R1 (Lixoft, Antony, France). A two-compartment model with two first-order elimination rates and an absorption compartment was implemented to describe the concentrations measured in serum and CSF. Proportional error models were used to describe residual variabilities in serum and CSF concentrations. Brain distribution of trastuzumab and anti-HER2 Fab#2 were assessed comparing typical values of parameters describing CSF-to-serum flow and elimination in CSF for each antibody.

**Statistical analysis**

For the comparison of the means, we used the Student T-test (2 means) for series distributed according to the normal distribution. To compare quantitative variables from multiple samples in the pharmacokinetic analysis, we used a multiparametric ANOVA test. All statistical analyses were performed using R software.

**Results**

**FcRn receptor is expressed by endothelial cells of the blood-brain barrier in human and rat brain**

FcRn is a transmembrane protein composed of two subunits: a large transmembrane alpha chain with 3 extracellular domains (α1, α2, α3), also called the subunit P51, and the β2 microglobulin subunit (28) (Supplementary Figure 1A). FcRn is ubiquitously expressed across different cells, tissues and species, including endothelial cells of the mammalian BBB (28). Using the Uniprot database, we compared the amino acid sequence of human, mouse and rat FcRn (Supplementary Figure 1B). Between human and rat, the homology is 67.1% for the entire protein composed of 484 amino acids. It is 64.6% for the P51 subunit and 69.7% for the β2microglobulin subunit. In contrast, the IgG binding site (green box on Figure 1B) contains amino-acids that are highly conserved between species (29).

On formalin-fixed brains from *Rattus norvegicus*, using immunostaining, we confirmed that the FcRn receptor is expressed at the meningeal (arachnoid and pie matter mainly) (Supplementary Figure 1C, low magnification), and in the choroid plexus epithelium (data not shown). At higher magnification, we showed a diffuse staining of brain meningeal and vessels, and that the FcRn receptor is preferentially located at the abluminal side of endothelial cells (Supplementary Figure 1C, X1000 magnification). When we assessed FcRn expression on human brains obtained from autopsies, we found a strong FcRn staining mainly located at the abluminal side of endothelial cells of arterioles and venules (Supplementary Figure 1D).

**No efflux from brain to blood after intra-CSF injection of a Fab in rats**

We performed a proof-of-concept study using a commercialized Fab ranibizumab, which is an anti-VEGF Fab antibody fragment, and bevacizumab, its corresponding full IgG1 antibody.

We first developed an ultra-sensitive ELISA to measure low concentrations of ranibizumab in a small volume of 10 µL. We applied a methodology developed by the Tours University Hospital to assess monoclonal antibody concentrations in patients. We first determined and validated a reproducible calibration curve of ranibizumab concentrations from 0 to 25 mg/L, and quality controls. All calibration points and quality controls were validated with a good reproducibility (assessed by a coefficient of variation <20%) and good accuracy (bias from expected concentrations <20%) (Supplementary Figure 2A and 2B). The limit of detection is 0.003 mg/L, the lower limit of quantification is 0.01 mg/L and the upper limit of quantification is 8.75 mg/L. The limit of sample dilution, also called dilution linearity is up to 2,000-fold.

After implantation of catheters into the cisterna magna of rats (Supplementary Figure 3A), we administered either bevacizumab or ranibizumab at equivalent concentrations (14 µg at 10 mg/mL for both solutions) in a series of 5 rats of each (Supplementary Figure 3B). Both bevacizumab and ranibizumab were detected in the CSF up to 240 minutes after the injection. As expected, bevacizumab was detected in the blood as early as 30 minutes after the injection with a gradual increase in concentration up to 360 minutes. In contrast, ranibizumab was never detected in the blood, suggesting an absence of early clearance from CSF to blood (Supplementary Figure 3C).

Using immunofluorescence staining on whole brains obtained at the time of euthanasia at 360 minutes, ranibizumab was identified in the cerebellum close to Purkinje cells (Supplementary Figure 3D), a region where VEGF is physiologically expressed and acts as a factor of neurogenesis and neuronal maturation (30).

**Engineering of anti-HER2 Fab antibodies**

*Anti-HER2 Fab synthesis*

In order to specifically target HER2-overexpressing breast cancer brain metastases, two different anti-HER2 Fab fragments of trastuzumab have been engineered using a comparable synthesis approach. The sequence was determined using the known Fab sequence of trastuzumab. The first fragment (anti-HER2 Fab#1) was produced by BIOTEM® Industry and initially led to 1.82 mg of a 45 kDa purified antibody. The analysis by SDS-PAGE under reducing conditions identified two fragments of ~23 kDa and ~25 kDa, corresponding respectively to the light and heavy chains of the Fab (Figure 1A, left panel). The protein sequence of the Fab fragment was then analyzed using mass spectrometry leading to a 94% sequence recognition. Analysis by SDS-PAGE under non-reducing conditions identified a fragment ~ 45 kDa, corresponding to Fab in its complete form (Figure 1A, right panel). The second Fab fragment (anti-HER2 Fab#2) was produced in our research unit (MASCOT) and extracted from the culture supernatant. The structure of the product fragment is a Fab because we identified two ~25 kDa fragments in SDS-PAGE (data not shown), with a kappa light chain identified on western-blot (Supplementary Figure 4A).

**Anti-HER2 Fab antibodies have *in vitro* effects comparable to those of trastuzumab**

To confirm that both anti-HER2 Fab antibodies efficiently bound HER2 membrane receptor, we used a fluorescent-labeled Fab to stain HER2-overexpressing BT474 cells. Triple negative MDA231 breast cancer cells were used as control. We observed a membrane labeling of 100% BT414 cells with trastuzumab as well as for the two Fab antibodies (anti-HER2 Fab#1 or #2) (Figure 1B). In all cases, MDA231 cells were not stained.

Using flow cytometry, we found no significant difference between anti-HER2 Fab antibodies and trastuzumab binding to cells at equivalent ponderal concentration (*P* = 0.88) (Supplementary Figure 4B). However, we observed that the Fab antibodies had a lower affinity (by a factor of 3) than trastuzumab avidity at the equivalent molarity concentration, as expected.

Therefore, in our study, we used a weight-based approach to assess the equivalence of therapeutic effects between the two antibody formats. We first assessed cytotoxic effect of the two anti-HER2 Fab antibodies on BT-474 cells and found an IC50 at 8 µg/mL identical to that of trastuzumab for the two Fab. For all concentrations tested, the two curves were superimposed (*P* = 0.46) (Supplementary Figure 4C). Since trastuzumab has a well-known cytostatic effect on cancer cells (31), we did an inhibition proliferation test over 4 days on BT-474 cells at the concentration of 8 µg/mL and found a similar decrease in cell proliferation with the two Fab (-63% for trastuzumab and -62% for the two Fab, *P* = 0.9) (Supplementary Figure 4D). For MDA-231 cells treated with trastuzumab or with the anti-HER2 Fab antibodies at the IC50, there was no inhibition proliferation effect.

**Anti-HER2 Fab antibodies have *in vivo* effects comparable to those of trastuzumab**

For this experiment, we used a patient derived xenograft model of HER2 overexpressing breast cancer, itself obtained from a skin metastasis of a patient sensitive to anti-HER2 treatments (18). We confirmed that the tumor xenograft overexpressed HER2 using immunochemistry (Supplementary Figure 5, left panel), with a very high *HER2* gene copy number of 28 (Supplementary Figure 5, right panel) which is highly predictive to response to anti-HER2 (32).

We first assessed anti-tumor effects of Fab#1, Fab#2 antibodies or trastuzumab administered intravenously in xenografted mice, at the weekly dose of 2 mg/kg, consensual for trastuzumab (33). We treated 10 animals per group. After 3 weeks of treatment, we observed a significant tumor growth inhibition compared to untreated mice for each of the three drugs (*P* < 0.001). There was no significant difference between the three anti-HER2 antibodies (Figure 1C). To study their tissue effects, we assessed necrosis, proliferation, apoptosis and microvessel density in the tumors. We found a direct effect on cancer cells with significant inhibition of proliferation in treated mice (Figure 1D). The Ki67 index was 37% ±6, 37% ±4, 39% ±9 and 51.5% ±8 respectively for mice treated with Fab#1, Fab#2, trastuzumab and for untreated mice (*P* <0.001). There was no significant difference between the two anti-HER2 Fab antibodies and trastuzumab (*P* = 0.3). We also found a significant decrease in microvessel density in viable areas of tumors for mice treated with trastuzumab or with the anti-HER2 Fab antibodies, compared to untreated mice (Figure 1E). Mean count of CD31-expressing vessels/HPF was 5.5 ±0.9, 5.4 ±0.4, 5.6 ±0.2, and 10.3 ±1.3 respectively for anti-HER2 Fab antibodies, trastuzumab and untreated mice (*P* < 0.001). No significant difference was observed between anti-HER2 Fab antibodies and trastuzumab (*P* = 0.8). Strikingly, this decrease in microvessel density was not associated with a significant increase in necrotic area (in yellow, Figure 1F), suggesting a neoangiogenesis inhibition with trastuzumab or the anti-HER2 Fabs, and not a direct cytotoxic effect on tumor endothelial cells. Tumor cell apoptosis, assessed using cleaved-caspase 3 immunostaining, was very sparse both in treated and untreated mice (data not shown).

A comparative pharmacological study was conducted on blood samples obtained before each intravenous injection (at Day 0, Day 7, Day 14 and Day 21), and 30 minutes after the first injection. Mean concentrations at 30 minutes were 45 mg/L and 10 mg/L respectively for trastuzumab and for the Fab#1. Trastuzumab steady-state concentration was of 1 mg/L while undetectable for the Fab#1 (Supplementary Figure 6). Trastuzumab half-life was estimated at 1.5 days. We could not calculate it for the Fab#2, probably because its serum half-life in mice is very short and because we did not have enough sampling between 30 minutes and Day 7.

Overall, anti-HER2 Fab antibodies had *in vitro* and *in vivo* anti-tumor effects comparable to those of trastuzumab.

**Anti-HER2 Fab antibodies and trastuzumab have no major cardiac toxicity**

When we assessed drug toxicity on normal tissues, including heart, kidney, liver and bone marrow, no damage was detected microscopically (data not shown). We particularly focused on cardiac toxicity, the only toxicity of trastuzumab. On frozen hearts obtained at the time of euthanasia, we assessed murine mRNA expression of two serum biomarkers of cardiac injury, *BNP* (a stress oxidant marker) and *Adrenomedullin* (an endothelial marker). *BNP* mRNA expression was only overexpressed in trastuzumab group (*P* < 0.05, Supplementary Figure 7A). We found a significant increase in mRNA expression of *Adrenomedullin*, both for trastuzumab and the Fab#2 when compared to untreated mice (*P* < 0.05, Supplementary Figure 7B). Using western-blotting for dipeptidyl-peptidase 3 (DPP3) (Supplementary Figure 7C) and cleaved-caspase3 (Supplementary Figure 7D), two markers of acute cardiac injury, we did not find significant difference between treated and untreated mice (*P* = 0.8 and *P* = 1).

**The anti-HER2 Fab antibody does not efflux from brain to blood after intraventricular injection in rats.**

To reduce the number of rats, we did pharmacological experiments with one of the two fragments (anti-HER2 Fab#1) because the anti-tumor effect of the two fragments was identical as we demonstrated. We first implemented the previously described ELISA procedure to detect low concentrations for trastuzumab and anti-HER2 Fab antibody in murine fluids*.* All calibration points and quality controls (QC) were validated with a good reproducibility and good accuracy (Supplementary Figure 8A and 8B). The lower limit of quantification was 0.1 mg/L for the Fab and 0.2 mg/L for trastuzumab.

At tissue level using standard histological colorations, we did not detect any brain toxicity after we injected 36.5 μg of trastuzumab or Fab#1, corresponding to the maximal dose of Fab injected in a volume of 100 µL. In a series of 10 rats, a pharmacokinetic study was conducted from T_0_ until one week. After intraventricular administration, the mean CSF concentration of trastuzumab reached 6.9 mg/L versus 3.0 mg/L for the Fab#1, at four hours. For the two drugs, CSF concentrations rapidly decreased from 4h to 48h, undetectable at 72h (Figure 2A). As expected, from 4h after intraventricular injection, we observed a rapid CSF-to-blood efflux of trastuzumab but not of the Fab#1 with no detection of the Fab#1 in blood (*P* = 0.01) (Figure 2B).

As the CSF concentration assayed in these in vivo treatment with trastuzumab or the Fab were low (14, 17, 18), we then decided to prepare a 50 times more concentrated Fab#1 and 7 additional rats were injected. Thirty minutes after intraventricular injection, mean peak concentrations were of 93.3 mg/L for trastuzumab and 32.5 mg/L for the Fab#1 as expected (Figure 2C). Then, trastuzumab CSF concentrations rapidly decreased and was no longer detected at 4h, with a serum concentration of 26.9 mg/L at 4h. In contrast, Fab#1 concentrations remained stable in CSF until 4h and were barely detected in serum (1.9 mg/L at 4h) (*P* = 0.002, Figure 2D). In addition, one hour after CSF administration, the Fab#1 was almost undetectable in normal lung, liver or kidney (Supplementary Figure 9).

We assessed the CSF-to-blood ratio calculated as [blood concentration] / [CSF concentration] (%) on the pooled data obtained from each of the 17 rats receiving trastuzumab or Fab#1. Efflux ratio steadily increased from 20% at 30 min to 2000% at 72h for trastuzumab. For the Fab#1, it was much lower, only reaching 150% at 72h (*P* < 0.001) (Figure 2E).

**The anti-HER2 Fab antibody penetrates into the deeper brain parenchyma**

Then, to better address the question of CSF-to-blood antibody efflux, we created a two-compartment pharmacokinetic model using a population approach (Figure 2F). We named k_10_ as the diffusion constant from CSF to brain, k_12_ as the diffusion constant from CSF to blood, and k_20_ as the elimination from serum constant. After CSF-to-blood efflux, the serum half-life in rats (calculated as Ln(2)/k_20_) was of 18 minutes for the Fab#1, and 1.9 days for trastuzumab (Table 1), in accordance with the half-life of trastuzumab calculated after intravenous injection in mice. The k_12_/k_10_ ratio was two-fold higher for trastuzumab than for the Fab#1, suggesting a greater brain penetration of the Fab#1. Independently from the model, we used data from 10 brain samples obtained at euthanasia to calculate the partition coefficient K_pu,ubrain_ as follow (Kp_u,ubrain_ = AUC_u_,_brain_ /AUC_u_,_csf_), extrapolated from a validated pharmacological parameter for the brain exposure to xenobiotics administered intravenously (34, 35). The Kp_u,ubrain_ was 12.3% for trastuzumab and 22.7% for Fab#1 (Figure 2G), in accordance with the result of the modeling approach. In particular, when we analyzed separately three parts of the brains (CE1, CE2 and CE3) (Figure 2H), the Kp_u,ubrain_ was 2.7 times higher for the Fab#1 than for trastuzumab in deeper brain area (CE1) (Table 1).

**Discussion**

In this preclinical study, we successfully engineered an anti-HER2 Fab and demonstrated its safety and equal efficacy to trastuzumab for the treatment of HER2 overexpressing breast cancer brain metastases.

The BIOTEM® industry, which carried out the synthesis for one of the two fragments (Fab#1), has ISO certification for the development and production of antibodies. We did not show any limiting toxicity in murine models except minor cardiac effect, well-known in the clinics and easily manageable for trastuzumab (36). In particular, there was no brain toxicity after intra-CSF administration. In addition, we confirmed the identical anti-tumor effect of the anti-HER2 fragments with trastuzumab in a relevant sub-cutaneous xenograft model, that could be confirmed using intracranial models of breast cancer brain metastases. The cell effects were mainly proliferation inhibition and inhibition of neo-angiogenesis, as previously reported for trastuzumab (37). Indeed, the binding of the antibody to the extra-cellular portion of HER2 receptor leads to cytostatic but also direct cytotoxic effects due to the inhibition of HER2 shedding inducing an inhibition of PI3K-AKT pathway, and attenuation of cell signaling (38). The role of the Fc portion in the cytotoxicity of therapeutic antibodies is widely described and supposed to rely mainly on antibody-dependent cell-mediated cytotoxicity (ADCC). Not to avoid the potential ADCC effect, we purposely used patient-derived xenografts in Rj:NMRI-Foxn1nu/nu mice with conserved innate immunity and functional NK (39). The antitumoral effects we observed were the same with the Fab and with trastuzumab suggesting that ADCC is a secondary effect in our highly HER2-expressing model. Nonetheless, recent engineering of anti-HER2 antibodies have been developed with modification of Fc linking FcRγIIIa to improve ADCC (40, 41).

Our pharmacokinetic study is original since it is the first study of intra-CSF administration of a Fab antibody, with the demonstration that such an antibody fragment, with this administration mode, was appropriate to limit the CSF-to-blood efflux and to increase brain parenchymal penetration of the drug. This was made possible by the implementation of original rat models with the implantation of catheters enabling repeated CSF sampling over time. After a unique injection, we limited the pharmacokinetic study to one week because the turnover of CSF is complete in 2.7 hours in rats, the CSF being completely renewed 9 times in 24 hours compared to 4 times in humans (42). The CSF-to-blood efflux of our anti-HER2 Fab was 5 to 15 times less marked than trastuzumab efflux which was massive from 30 minutes after intraventricular administration, suggesting the hypothesis of a main efflux of this therapeutic IgG by the FcRn receptor of the BBB. This decreased efflux of the fragment antibodies, with no Fc, has also been described with the anti-VEGF Fab ranibizumab when injected in the corpus vitreum of cynomolgus monkeys. Interestingly, the authors observed a low blood efflux with serum concentrations 1500-times lower than in the vitreous compartment (43), finally similar to what we observed in the brain, which is consistent with the fact that the blood-retinal barrier is composed of tight junctions thus mimicking the BBB structure (44). From our pharmacokinetic model parameters, we demonstrated that the low CSF-to-blood efflux of our anti-HER2 Fab was associated with a higher brain penetration, by two-fold compared to trastuzumab. When administered intravenously, Fab antibodies have much faster clearances but also much larger distribution volumes (45, 46), justifying specification formulations like PEGylation to increase their blood half-life (47). After intraventricular administration, our Fab was barely detected in compartments outside the central nervous system, suggesting limited active efflux systems. Like for xenobiotics, it probably diffused *via* the glymphatic system into the cerebral interstitium by convective system, before later elimination into the venous circulation (20). Interestingly, despite a complete CSF turnover in 2.7 hours in rats, the Fab was still detected in CSF at 72h after a unique injection, suggesting that its elimination through the normal venous circulation is slow, and that the Fab may accumulate into the interstitium, partly owing to a lack of FcRn-linked detoxification mechanism. Indeed, our anti-HER2 Fab can penetrate deeper brain parenchyma, which shall be explained by its smaller size. Whether the Fab antibody may be locally degraded remains unclear, and we would need longer cerebral and serum pharmacokinetics data to determine the fate of the molecule.

A limitation of the intraventricular approach is its difficult acceptance in clinical practice. It requires intraventricular catheterization of the CSF under general anesthesia, and all injections must be performed under strict aseptic conditions. Thanks to the Fab design, we will be able to considerably space the time between injections and thus propose a lumbar route of administration (48), to improve the quality of life of patients in resort situations. Consideration could also be given to designing a formulation to further increase the half-life of Fab in the CSF and penetration into the brain parenchyma. Additional ways of administration such as the intra-nasal route could also be considered with our Fab (49, 50), pending brain parenchyma penetration at pharmacologically relevant concentrations.

Finally, the original concept of Fab fragments of monoclonal antibodies is very promising in view of the development of therapeutic antibodies in many indications, especially in the field of central nervous system pathologies including other malignancies but also neurodegenerative diseases (51, 52).

**Conclusions**

In conclusion, we successfully engineered and did the preclinical development of a trastuzumab Fab as effective as its native IgG, and capable of doubling brain penetration and significantly reducing CSF-to-blood efflux after intra-CSF injection. This Fab could thus be a new and original effective drug in the treatment of HER2 breast cancer brain metastases, which should be demonstrated by a phase I clinical trial dedicated to women in resort situations.

**References**

1. Achrol AS, Rennert RC, Anders C, Soffietti R, Ahluwalia MS, Nayak L, et al. Brain metastases. Nat Rev Dis Primers. 2019;5(1):5.

2. Cagney DN, Martin AM, Catalano PJ, Redig AJ, Lin NU, Lee EQ, et al. Incidence and prognosis of patients with brain metastases at diagnosis of systemic malignancy: a population-based study. Neuro Oncol. 2017;19(11):1511-21.

3. Baselga J, Cortes J, Kim SB, Im SA, Hegg R, Im YH, et al. Pertuzumab plus trastuzumab plus docetaxel for metastatic breast cancer. N Engl J Med. 2012;366(2):109-19.

4. Swain SM, Miles D, Kim SB, Im YH, Im SA, Semiglazov V, et al. Pertuzumab, trastuzumab, and docetaxel for HER2-positive metastatic breast cancer (CLEOPATRA): end-of-study results from a double-blind, randomised, placebo-controlled, phase 3 study. Lancet Oncol. 2020;21(4):519-30.

5. Hall WA, Djalilian HR, Nussbaum ES, Cho KH. Long-term survival with metastatic cancer to the brain. Med Oncol. 2000;17(4):279-86.

6. Lin NU, Winer EP. Brain metastases: the HER2 paradigm. Clin Cancer Res. 2007;13(6):1648-55.

7. Angeli E, Nguyen TT, Janin A, Bousquet G. How to Make Anticancer Drugs Cross the Blood-Brain Barrier to Treat Brain Metastases. Int J Mol Sci. 2019;21(1).

8. Li J, Jiang J, Bao X, Kumar V, Alley SC, Peterson S, et al. Mechanistic Modeling of Central Nervous System Pharmacokinetics and Target Engagement of HER2 Tyrosine Kinase Inhibitors to Inform Treatment of Breast Cancer Brain Metastases. Clin Cancer Res. 2022;28(15):3329-41.

9. Lin NU, Borges V, Anders C, Murthy RK, Paplomata E, Hamilton E, et al. Intracranial Efficacy and Survival With Tucatinib Plus Trastuzumab and Capecitabine for Previously Treated HER2-Positive Breast Cancer With Brain Metastases in the HER2CLIMB Trial. J Clin Oncol. 2020;38(23):2610-9.

10. Murthy RK, Loi S, Okines A, Paplomata E, Hamilton E, Hurvitz SA, et al. Tucatinib, Trastuzumab, and Capecitabine for HER2-Positive Metastatic Breast Cancer. N Engl J Med. 2020;382(7):597-609.

11. Bartsch R, Berghoff AS, Furtner J, Marhold M, Bergen ES, Roider-Schur S, et al. Trastuzumab deruxtecan in HER2-positive breast cancer with brain metastases: a single-arm, phase 2 trial. Nat Med. 2022;28(9):1840-7.

12. Modi S, Saura C, Yamashita T, Park YH, Kim SB, Tamura K, et al. Trastuzumab Deruxtecan in Previously Treated HER2-Positive Breast Cancer. N Engl J Med. 2020;382(7):610-21.

13. Jerusalem G, Park YH, Yamashita T, Hurvitz SA, Modi S, Andre F, et al. Trastuzumab Deruxtecan in HER2-Positive Metastatic Breast Cancer Patients with Brain Metastases: A DESTINY-Breast01 Subgroup Analysis. Cancer Discov. 2022;12(12):2754-62.

14. Bonneau C, Paintaud G, Tredan O, Dubot C, Desvignes C, Dieras V, et al. Phase I feasibility study for intrathecal administration of trastuzumab in patients with HER2 positive breast carcinomatous meningitis. Eur J Cancer. 2018;95:75-84.

15. Zagouri F, Sergentanis TN, Bartsch R, Berghoff AS, Chrysikos D, de Azambuja E, et al. Intrathecal administration of trastuzumab for the treatment of meningeal carcinomatosis in HER2-positive metastatic breast cancer: a systematic review and pooled analysis. Breast Cancer Res Treat. 2013;139(1):13-22.

16. Kumthekar PU, Avram MJ, Lassman AB, Lin NU, Lee E, Grimm SA, et al. A phase I/II study of intrathecal trastuzumab in human epidermal growth factor receptor 2-positive (HER2-positive) cancer with leptomeningeal metastases: Safety, efficacy, and cerebrospinal fluid pharmacokinetics. Neuro Oncol. 2023;25(3):557-65.

17. Bousquet G, Darrouzain F, de Bazelaire C, Ternant D, Barranger E, Winterman S, et al. Intrathecal Trastuzumab Halts Progression of CNS Metastases in Breast Cancer. J Clin Oncol. 2016;34(16):e151-5.

18. Nguyen TT, Angeli E, Darrouzain F, Nguyen QT, Desvignes C, Rigal M, et al. A successful compartmental approach for the treatment of breast cancer brain metastases. Cancer Chemother Pharmacol. 2019;83(3):573-80.

19. Zhang Y, Pardridge WM. Mediated efflux of IgG molecules from brain to blood across the blood-brain barrier. J Neuroimmunol. 2001;114(1-2):168-72.

20. Paris J, Angeli E, Bousquet G. The Pharmacology of Xenobiotics after Intracerebro Spinal Fluid Administration: Implications for the Treatment of Brain Tumors. International journal of molecular sciences. 2021;22(3).

21. Loscher W, Potschka H. Drug resistance in brain diseases and the role of drug efflux transporters. Nat Rev Neurosci. 2005;6(8):591-602.

22. Schinkel AH, Wagenaar E, Mol CA, van Deemter L. P-glycoprotein in the blood-brain barrier of mice influences the brain penetration and pharmacological activity of many drugs. J Clin Invest. 1996;97(11):2517-24.

23. Ternant D, Ceze N, Lecomte T, Degenne D, Duveau AC, Watier H, et al. An enzyme-linked immunosorbent assay to study bevacizumab pharmacokinetics. Ther Drug Monit. 2010;32(5):647-52.

24. Durand M, Oger M, Nikovics K, Venant J, Guillope AC, Jouve E, et al. Influence of the Immune Microenvironment Provided by Implanted Biomaterials on the Biological Properties of Masquelet-Induced Membranes in Rats: Metakaolin as an Alternative Spacer. Biomedicines. 2022;10(12).

25. Percie du Sert N, Hurst V, Ahluwalia A, Alam S, Avey MT, Baker M, et al. The ARRIVE guidelines 2.0: Updated guidelines for reporting animal research. PLoS Biol. 2020;18(7):e3000410.

26. Bustin SA, Benes V, Garson JA, Hellemans J, Huggett J, Kubista M, et al. The MIQE guidelines: minimum information for publication of quantitative real-time PCR experiments. Clinical chemistry. 2009;55(4):611-22.

27. Bernadou G, Campone M, Merlin JL, Gouilleux-Gruart V, Bachelot T, Lokiec F, et al. Influence of tumour burden on trastuzumab pharmacokinetics in HER2 positive non-metastatic breast cancer. Br J Clin Pharmacol. 2016;81(5):941-8.

28. Latvala S, Jacobsen B, Otteneder MB, Herrmann A, Kronenberg S. Distribution of FcRn Across Species and Tissues. J Histochem Cytochem. 2017;65(6):321-33.

29. Huang X, Zheng F, Zhan CG. Binding structures and energies of the human neonatal Fc receptor with human Fc and its mutants by molecular modeling and dynamics simulations. Mol Biosyst. 2013;9(12):3047-58.

30. Licht T, Keshet E. Delineating multiple functions of VEGF-A in the adult brain. Cell Mol Life Sci. 2013;70(10):1727-37.

31. Argiris A, Wang CX, Whalen SG, DiGiovanna MP. Synergistic interactions between tamoxifen and trastuzumab (Herceptin). Clin Cancer Res. 2004;10(4):1409-20.

32. Gonullu B, Angeli E, Pamoukdjian F, Bousquet G. HER2 Amplification Level Predicts Pathological Complete Response in the Neoadjuvant Setting of HER2-Overexpressing Breast Cancer: A Meta-Analysis and Systematic Review. Int J Mol Sci. 2023;24(4).

33. Slamon DJ, Leyland-Jones B, Shak S, Fuchs H, Paton V, Bajamonde A, et al. Use of chemotherapy plus a monoclonal antibody against HER2 for metastatic breast cancer that overexpresses HER2. N Engl J Med. 2001;344(11):783-92.

34. Di L, Riccardi K, Tess D. Evolving approaches on measurements and applications of intracellular free drug concentration and Kp(uu) in drug discovery. Expert Opin Drug Metab Toxicol. 2021;17(7):733-46.

35. Loryan I, Reichel A, Feng B, Bundgaard C, Shaffer C, Kalvass C, et al. Unbound Brain-to-Plasma Partition Coefficient, K(p,uu,brain)-a Game Changing Parameter for CNS Drug Discovery and Development. Pharm Res. 2022;39(7):1321-41.

36. Jerusalem G, Lancellotti P, Kim SB. HER2+ breast cancer treatment and cardiotoxicity: monitoring and management. Breast Cancer Res Treat. 2019;177(2):237-50.

37. Izumi Y, Xu L, di Tomaso E, Fukumura D, Jain RK. Tumour biology: herceptin acts as an anti-angiogenic cocktail. Nature. 2002;416(6878):279-80.

38. Valabrega G, Montemurro F, Aglietta M. Trastuzumab: mechanism of action, resistance and future perspectives in HER2-overexpressing breast cancer. Ann Oncol. 2007;18(6):977-84.

39. Radaelli E, Santagostino SF, Sellers RS, Brayton CF. Immune Relevant and Immune Deficient Mice: Options and Opportunities in Translational Research. ILAR J. 2018;59(3):211-46.

40. Rugo HS, Im SA, Cardoso F, Cortes J, Curigliano G, Musolino A, et al. Efficacy of Margetuximab vs Trastuzumab in Patients With Pretreated ERBB2-Positive Advanced Breast Cancer: A Phase 3 Randomized Clinical Trial. JAMA Oncol. 2021;7(4):573-84.

41. Schram AM, Odintsov I, Espinosa-Cotton M, Khodos I, Sisso WJ, Mattar MS, et al. Zenocutuzumab, a HER2xHER3 Bispecific Antibody, Is Effective Therapy for Tumors Driven by NRG1 Gene Rearrangements. Cancer Discov. 2022;12(5):1233-47.

42. Barten DM, Cadelina GW, Weed MR. Chapter 4 - Dosing, collection, and quality control issues in cerebrospinal fluid research using animal models2018.

43. Gaudreault J, Fei D, Rusit J, Suboc P, Shiu V. Preclinical pharmacokinetics of Ranibizumab (rhuFabV2) after a single intravitreal administration. Invest Ophthalmol Vis Sci. 2005;46(2):726-33.

44. Cunha-Vaz J, Bernardes R, Lobo C. Blood-retinal barrier. Eur J Ophthalmol. 2011;21 Suppl 6:S3-9.

45. Covell DG, Barbet J, Holton OD, Black CD, Parker RJ, Weinstein JN. Pharmacokinetics of monoclonal immunoglobulin G1, F(ab')2, and Fab' in mice. Cancer Res. 1986;46(8):3969-78.

46. Rafidi H, Rajan S, Urban K, Shatz-Binder W, Hui K, Ferl GZ, et al. Effect of molecular size on interstitial pharmacokinetics and tissue catabolism of antibodies. MAbs. 2022;14(1):2085535.

47. Jevsevar S, Kusterle M, Kenig M. PEGylation of antibody fragments for half-life extension. Methods Mol Biol. 2012;901:233-46.

48. Lazaratos AM, Maritan SM, Quaiattini A, Darlix A, Ratosa I, Ferraro E, et al. Intrathecal trastuzumab versus alternate routes of delivery for HER2-targeted therapies in patients with HER2+ breast cancer leptomeningeal metastases. Breast. 2023;69:451-68.

49. Chen TC, da Fonseca CO, Schonthal AH. Intranasal Perillyl Alcohol for Glioma Therapy: Molecular Mechanisms and Clinical Development. Int J Mol Sci. 2018;19(12).

50. Gomes MJ, Fernandes C, Martins S, Borges F, Sarmento B. Tailoring Lipid and Polymeric Nanoparticles as siRNA Carriers towards the Blood-Brain Barrier - from Targeting to Safe Administration. J Neuroimmune Pharmacol. 2017;12(1):107-19.

51. van Dyck CH, Swanson CJ, Aisen P, Bateman RJ, Chen C, Gee M, et al. Lecanemab in Early Alzheimer's Disease. N Engl J Med. 2023;388(1):9-21.

52. Cavaco M, Gaspar D, Arb Castanho M, Neves V. Antibodies for the Treatment of Brain Metastases, a Dream or a Reality? Pharmaceutics. 2020;12(1).
